# Shadow estimation of gate-set properties from random sequences

**DOI:** 10.1038/s41467-023-39382-9

**Published:** 2023-08-19

**Authors:** J. Helsen, M. Ioannou, J. Kitzinger, E. Onorati, A. H. Werner, J. Eisert, I. Roth

**Affiliations:** 1 QuSoft, Centrum Wiskunde & Informatica (CWI), Amsterdam, The Netherlands; 2 Korteweg-de Vries Institute for Mathematics, University of Amsterdam, Amsterdam, The Netherlands; 3 Dahlem Center for Complex Quantum Systems, Freie Universität Berlin, 14195 Berlin, Germany; 4 Humboldt-Universität zu Berlin, Institut für Physik, 12489 Berlin, Germany; 5 Department of Computer Science, University College London, London, UK; 6 Fakultät für Mathematik, Technische Universität München, München, Germany; 7 Department of Mathematical Sciences, University of Copenhagen, 2100 København, Denmark; 8 NBIA, Niels Bohr Institute, University of Copenhagen, Blegdamsvej 17, 2100 København, Denmark; 9 Helmholtz-Zentrum Berlin für Materialien und Energie, 14109 Berlin, Germany; 10 Fraunhofer Heinrich Hertz Institute, 10587 Berlin, Germany; 11 Quantum Research Center, Technology Innovation Institute (TII), Abu Dhabi, UAE

**Keywords:** Quantum information, Theoretical physics, Information theory and computation

## Abstract

With quantum computing devices increasing in scale and complexity, there is a growing need for tools that obtain precise diagnostic information about quantum operations. However, current quantum devices are only capable of short unstructured gate sequences followed by native measurements. We accept this limitation and turn it into a new paradigm for characterizing quantum gate-sets. A single experiment—random sequence estimation—solves a wealth of estimation problems, with all complexity moved to classical post-processing. We derive robust channel variants of shadow estimation with close-to-optimal performance guarantees and use these as a primitive for partial, compressive and full process tomography as well as the learning of Pauli noise. We discuss applications to the quantum gate engineering cycle, and propose novel methods for the optimization of quantum gates and diagnosing cross-talk.

## Abstract (web summary)

In order to be practical, schemes for characterizing quantum operations should require the simplest possible gate sequences and measurements. Here, the authors show how random gate sequences and native measurements (followed by classical post-processing) are sufficient for estimating several gate set properties.

## Introduction

Recent years have seen the rapid development of quantum computing devices to unprecedented system sizes. These devices are still noisy and of limited computational power, but go substantially beyond what was conceivable not very long ago. In order to scale even further to larger and more accurate devices, it is key to develop tools for efficiently characterizing quantum operations^1,2^ at scale. Besides providing crucial actionable advice for the practitioner, the characterization of quantum operations is also important for developing an in-depth theoretical understanding of the actual capabilities of quantum devices and for providing a fair comparison between different types of devices, and with classical computing power on the same tasks^3–5^. Over the years, many protocols for characterizing quantum operations have been developed^6–8^.

That said, while a wealth of theoretical ideas for benchmarking, verification, and tomographic recovery have been suggested, only a few of them are relevant in practice. With present quantum devices, only relatively short gate sequences can be implemented on qubit arrays, followed by a native measurement at the end of the circuit that typically suffers from sizeable read-out noise. With these limitations, the most prominent protocols for characterizing digital quantum gates fall into the class of randomized benchmarking (RB)^9–14^ (including newer protocols such as *averaged circuit eigenvalue sampling*^15^). RB implements suitable sequences of random quantum gates and extracts a measure of quality as parameters describing the decay rate of the measured signal with the sequence length. This has the advantage of yielding *state preparation and measurement* (SPAM) error robust error metrics. The experimental sequences of most RB protocols are carefully designed (such as compiled circuit inverses) to efficiently extract specific information from a gate set. Prominent exceptions are ‘filtered’ RB protocols such as *linear cross-entropy benchmarking* (XEB)^3^ that directly work with random sequences of i.i.d. drawn gates and, e.g., omit an inversion gate.

In this work, we take these observations seriously and revert to the mindset that is commonly applied when devising new schemes for benchmarking and characterization. We ask the question: If all we can feasibly do is implement unstructured random sequences followed by a native measurement, what can we learn? At first sight, this endeavor is not promising. Compared to ‘traditional’ RB and tomographic protocols we are giving up on central ingredients. Thinking about how much information we measure in an unstructured way, we run into the problem that typically, the probabilities of individual measurement results are exponentially small in the number of qubits. This is orthogonal to the careful design of efficient characterization schemes in prior work and does not obviously yield sample efficient estimation schemes at all.

Our change of paradigm is analogous to the mindset of classical shadows^16,17^. Classical state shadows allow for the sample-efficient estimation of (exponentially) many different functions of a quantum state from the same data by only modifying the classical post-processing. Perhaps the central surprise value of the result of ref. ^16^ is rigorously guaranteeing that the fidelity of a quantum state with respect to any pure state can be estimated from the same experiment, using only constantly many state copies with sufficiently randomized basis measurements. This is in stark contrast to schemes like direct fidelity estimation^18^ that given a priori knowledge of the target state carefully optimize the measurements that are performed.

In this work, we define the observed measurement outcomes of random sequences of quantum gates as the *classical shadow of a gate-set* and study the sample efficiency of SPAM-robust estimators for different linear functionals of a gate-set from the same data. Borrowing the median-of-means estimators used on classical state shadows, we show that the sampling complexity of the estimation (the number of single-shot quantum measurements) can be controlled by a dynamic shadow norm with exponential confidence. We prove bounds on this dynamic shadow norm—a considerably more involved object than its state counterpart—for prominent gate-sets such as the multi-qubit Clifford group and the local Clifford group. We find that by a suitable post-processing we can estimate the relative average gate fidelities of the noise of a Clifford gate-set with respect to an exponentially large number of unitary channels from polynomially many measurement samples from the same uniformly random experiment. More generally, we show that the dynamical shadow norm can be controlled in terms of the unitarity of the estimated linear quantity. Using local gate-sets, we show that one can selectively gain information about channel marginals capturing correlations in their noisy implementation. We promote this primitive further to design a highly scalable and efficient tomography scheme for cross-talk effects. Furthermore, we exemplify how gate-set shadows can be used to construct SPAM-robust objective functions for learning noise models and for robust low-rank quantum process tomography.

The important feature of all these schemes is that we only adopt the classical post-processing to the task at hand, not the quantum experiment. A single type of data, namely samples from simple local measurements on uniformly random gate sequences, is sufficient to perform a large class of diagnostic tasks of benchmarking, verification, and tomographic recovery. The mindset can be captured as “Measure first, ask later!”. Going beyond uniformly independently random sequences, we can generalize our approach to provide an optimal scheme to learn Pauli noise, emulating the protocol of ref. ^19^ with a simpler experimental prescription and theoretical analysis.

*Related work*: We build on a body of literature on randomized schemes for quantum device characterization^8^. The potential of analyzing the output statistics of gate-set sequences to self-consistently extract essentially all information of a gate-set (as well as the initial state and the measurement) has been realized by gate-set tomography^20–25^ with recent variants only requiring random sequences (gate-set shadows)^26,27^. In contrast to this self-consistent tomographic estimation of all gates in the gate-set, we here target individual linear quantities of the gate-set’s average noise or an interleaved quantum process. Our cross-talk tomography protocol follows the spirit of simultaneous RB^28^, but goes significantly beyond simultaneous RB in providing higher-order correlation measures and tomographic information of noise-channel marginals, efficiently from the data of a single randomized experiment. In ref. ^29^, it has been observed that variants of interleaved multi-qubit Clifford randomized benchmarking experiments^30^ have access to relative average gate fidelities from which unital quantum channels can be reconstructed. The protocol of ref. ^29^ performs a different experiment for each fidelity yielding a sub-optimal overall sample complexity for tomography or low-rank tomography^31,32^. Gate-set shadow estimation solves both these short-comings.

## Results

**Fig. 1:**
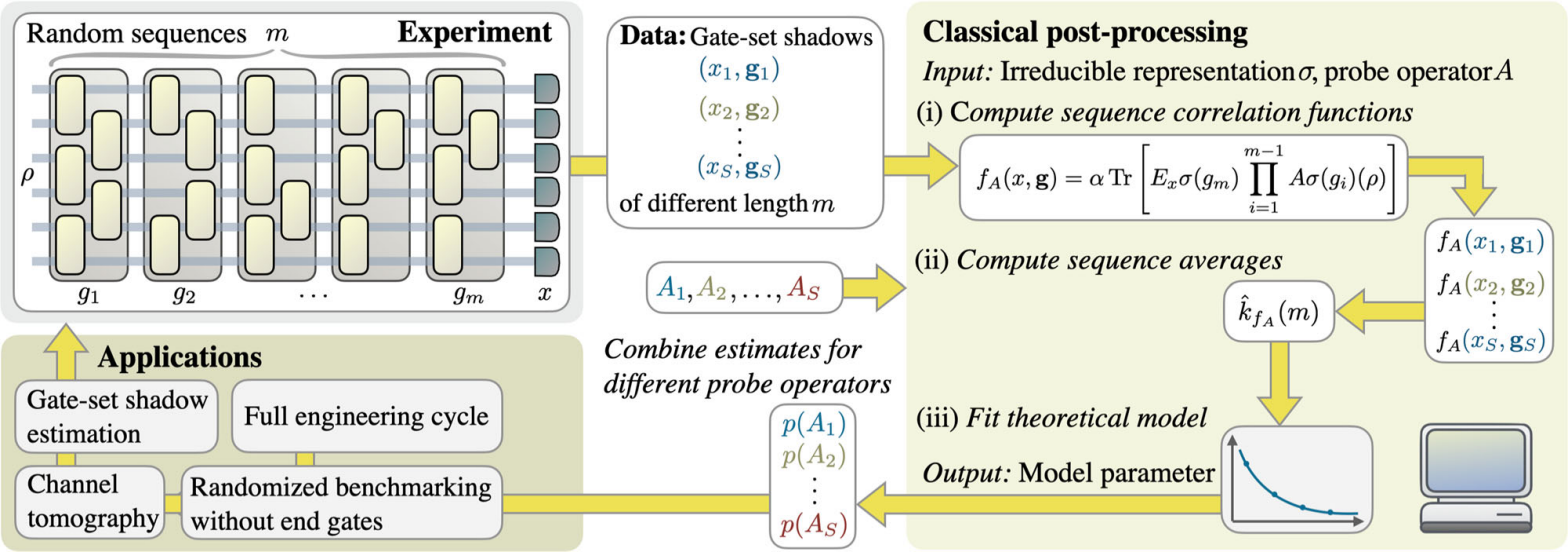
The gate-set shadow estimation protocol proceeds in two stages. First, for a fixed initial state *ρ* and varying sequence lengths *m* a total of *S* random sequences of quantum gates of length *m* are experimentally implemented and each is followed by a measurement. We call the observed tuples of measurement outcome and gate sequence $({x}^{j},\, {g}_{1}^{j} \,,\ldots,\, {g}_{m}^{j})$, *j* = 1, …, *S* the gate-set shadow. The second classical post-processing stage consists itself of three steps: (i) A given sequence correlation function is calculated for every entry of the gate set shadow. For the UIRS protocol a sequence correlation function *f*_*A*_ is specified in terms of a probe super-operator *A* and an irreducible representation *σ*. (ii) We calculate the sequence average ${\hat{k}}_{{f}_{A}}(m)$ as the mean or median-of-means of the result of step (i) over sequences of the same length *m*. (iii) Sequence averages for different lengths *m* are used as data points to fit a theoretical model (Eq. (5)) in order to extract the generalized gate-set fidelity with respect to the super-operator *A* and the irreducible representation *σ*, denoted here by *p*(*A*). One of the most important features of this approach is that we can use the same experimental data to accurately estimate *exponentially many* generalized fidelities *p*(*A*_1_), *p*(*A*_2_), …, *p*(*A*_S_) by evaluating different sequence correlation functions on the same gate-set shadow. In this way, we can self-consistently and robustly estimate many different properties of the gate-set noise from a minimal amount of data obtained in a simple experiment. Sections “Application: Learning unitary noise models”, “Application: Cross-talk tomography”, and “Application: SPAM-robust channel reconstruction” explain and derive guarantees of how the gate-set shadow estimation protocol can be used as a primitive in other more detailed characterization task, such as compressive channel or marginal tomography, potentially allowing one to run the whole engineering cycle on essentially the same type of data.

We begin with explaining the general protocol. In the subsequent sections, we then provide theoretical performance guarantees for specific gate-sets and explain how the protocol can be used as a robust estimation primitive in more complex characterization tasks, such as channel tomography. The gate-set shadow estimation protocol consists of two separate stages: an experiment, where measurement results from random circuits of different lengths are recorded, and a classical post-processing step, where different parameters can be estimated from the measured data. Figure 1 summarizes the complete protocol.

### Protocol: Experiment

We aim at characterizing the accuracy of the implementation of a target gate-set ${\mathbb{G}}$. The experimental primitive is the realization of random (gate) sequences of length *m*: After preparing an initial *ρ* (e.g., $\left|0\right\rangle \left\langle 0\right|$) a sequence of gates ${{{{\boldsymbol{g}}}}}\in {{\mathbb{G}}}^{\times m}$ is drawn at random according to a distribution ${\mu }_{m}:{{\mathbb{G}}}^{\times m}\to [0,1]$ and applied to *ρ*. This is then followed by a measurement specified by a POVM ${\{{E}_{x}\}}_{x}$ with measurement outcomes in ${{{{{{{\mathcal{X}}}}}}}}$ (e.g., a computational basis measurement). If $x\in {{{{{{{\mathcal{X}}}}}}}}$ is observed, the result of the primitive is a tuple $(x,\, {{{{\boldsymbol{g}}}}})\in {{{{{{{\mathcal{X}}}}}}}}\times {{\mathbb{G}}}^{\times m}$.

Repeating the primitive multiple times yields a series of tuples ${\{({x}_{i},\, {{{{{\boldsymbol{g}}}}}}_{i})\}}_{i=1}^{S}$ which we refer to as a *(self-consistent) gate-set shadow*. (Note that ref. ^16^ actually calls the dual frame elements indexed by the observed output statistics of an informationally complete POVM a state’s *shadow*. In contrast, we here directly refer to the sampled sequence and observed measurement outcomes as a shadow.)

A complete experimental protocol further involves measuring such shadows for a set of different sequence lengths *m*. In order to simplify the theoretical analysis, we focus on the paradigmatic case of ${\mathbb{G}}$ being a finite subgroup of SU(2^*N*^) (such as the Clifford group) and distributions on the sequences arising from the uniform measure over these subgroups.

The simplest example of protocols in this context is *uniform independent random sequence (or* UIRS) *protocols* where the gates in the sequences are drawn from the gate-set uniformly and independently at random. This can be seen as the paradigmatic case, although we will go beyond this later in this work. We make shadow gate-set estimation through the UIRS protocol explicit for several important gate-sets: namely the multi-qubit Clifford group ${{\mathbb{C}}}_{n}$ and the independent single-qubit Clifford group ${{\mathbb{C}}}_{1}^{\times n}$ (which we will call the *local Clifford group*).

### Protocol: Classical post-processing

Given a gate-set shadow ${\{({x}_{i},\, {{{{{\boldsymbol{g}}}}}}_{i})\}}_{i=1}^{S}$, we define an empirical estimator in terms of a *sequence correlation function *$f(x,\, {{{{{\boldsymbol{g}}}}}}):{{{{{{{\mathcal{X}}}}}}}}\times {{\mathbb{G}}}^{\times m}\to {\mathbb{C}}$. For every such sequence correlation function, in the post-processing, we (i) evaluate *f* for all entries of the gate-set shadows and (ii) calculate the empirical mean or median-of-means estimator

1 $${\hat{k}}_{f}(m): \!=\,{{\mbox{(median-of-)means}}} \, {\{f({x}_{i},\, {{{{{\boldsymbol{g}}}}}}_{i})\}}_{i=1}^{S}$$

of the result. After repeating steps (i) and (ii) for different sequence lengths *m*, we fit in step (iii) a theoretical model *k*_*f*_ to the estimates of the *sequence means* ${\hat{k}}_{f}(m)$. After giving this overview of the post-processing protocol, let us take a closer look at the steps and explain their roles in the UIRS protocol:

Regarding step (i): Generally speaking, sequence correlation functions can be seen as the gate-set analog of an observable in shadow estimation. They allow us to compute properties of noisy gate-sets (for example the average fidelity of an average group element) from experimentally observed gate-set shadows. We emphasize that, like state shadow estimation, the data collection step of random sequence estimation is independent of the gate-set properties one wishes to estimate, with this estimation step happening entirely in classical post-processing. Importantly, this enables one to estimate many different correlation functions from the same experimental data.

We here introduce a particular class of sequence correlation functions for UIRS protocols: Consider an irreducible representation *σ* of ${\mathbb{G}}$ with representation space *V*_*σ*_. For the multi-qubit Clifford group, e.g., its adjoint action on traceless Hermitian matrices is of main interest. We further specify a sequence correlation function in terms of a matrix *A*, POVM ${\{{E}_{x}\}}_{x\in {{{{{{{\mathcal{X}}}}}}}}}$ and state *ρ*, on *V*_*σ*_ as

2 $${f}_{A}(x,\, {{{{{\boldsymbol{g}}}}}})=\alpha \,{{{{{{{\rm{Tr}}}}}}}}\left[{E}_{x}\sigma ({g}_{m})\mathop{\prod }\limits_{i=1}^{m-1}A\sigma ({g}_{i})(\rho )\right]\,,$$

with a suitable normalization factor *α*. (Note that for *m* = 1, and perfectly implemented gates, this expression reproduces the classical state shadows of ref. ^16^. Generally, restricted to multiplicity-free, irreducible representations, the dual frame construction of ref. ^16^ simply amounts to introducing a proper normalization factor, justifying our choice of calling the observed statistics directly the shadow.)

We refer to *A* as a *probe (super-)operator* as it specifies the linear quantity of the gate-set that is encoded into the decay parameter of the empirical estimator. Note that the expression Eq. (2) is closely related to the Born probability of measuring *x* after applying the sequence ${{{{\boldsymbol{g}}}}}$ to *ρ*. The main differences are that we restrict the computation to the subspace *V*_*σ*_ and interleave the sequence with the probe operator *A*. Similar to classical shadows, the computation of *f*_*A*_ requires, in general, the same resources as simulating the physical evolution within a subspace. In many situations, however, further structure renders this task efficient. This is in particular the case when both the gate-set and the probe-operators are chosen to be multi-qubit Clifford operations.

Note that all previously existing RB protocols only use functions that at most depend on the product of the operations in the sequence, ${f}_{{\mathbb{I}}}(x,\, {{{{{\boldsymbol{g}}}}}})=h(x,\, {g}_{1}{g}_{2}\cdots {g}_{m})$. In filtered RB protocols, such as linear cross-entropy benchmarking^3^, character benchmarking^33^ and Pauli-noise tomography^19^, the inversion gate can in this way be omitted and accounted for in post-processing. Using a non-trivial *A* goes significantly beyond existing schemes and allows one to even efficiently ‘interleave in-post’ the same data with different probe operators.

Regarding step (ii): By taking an empirical average over the gate-set, we expect ${\hat{k}}_{f}(m)$ to be a degree *m* polynomial in the ‘average noise’ of the gate-set. One insight of standard Clifford randomized benchmarking is that by taking a uniform average over a sufficiently large group the ‘average noise’ is probed isotropically, effectively projecting it onto a depolarizing channel. Similarly, UIRS will probe the ‘average noise’ of the gate-set, but by choosing different probe operators *A*, we can alter the operator on which the noise is projected, revealing more information. Performing the post-processing separately for different irreducible representations *σ*, ensures that the gate-set always averages sufficiently over the subspace under consideration. We will make this intuition precise in the subsequent section.

Regarding step (iii): The projection onto isotropic noise (on each representation space) also dramatically ‘simplifies’ the functional form of the expected value of the sequence averages ${\hat{k}}_{f}(m)$. Recall that for standard Clifford RB, one effectively witnesses a single exponential decay. Below we show that analogously for UIRS protocols, the theoretical fitting model is a single (matrix) exponential decay encoding linear quantities of the noise in its decay parameter. The decay parameter(s) can be extracted using least-square fitting algorithms (or tone-finding algorithms such as ESPRIT). See ref. [^14^, Sec. VII] for a discussion on different post-processing techniques. In the end, the UIRS gate-set shadow estimation protocol returns the decay parameters for different choices of probe operators *A* and representations *σ*.

### Fitting model

In order to keep the theoretical derivation and statements concise and straightforward to interpret, we adhere to some standard assumptions that are commonly used in the analysis of RB protocols. First, we assume that the quantum channel that implements a sequence ${{{{{\boldsymbol{g}}}}}}$ on the quantum device can be written as ${{{{{{{\mathcal{E}}}}}}}}\,({{{{{\boldsymbol{g}}}}}})=\mathop{\prod }\nolimits_{i=1}^{m}\phi ({g}_{i})$ with a map $\phi :{\mathbb{G}}\to {{{{{{{{\mathcal{S}}}}}}}}}_{n}$. Here, ${{{{{{{{\mathcal{S}}}}}}}}}_{n}$ is the space of *n*-qubit super-operators. The existence of ${{{{{{{\mathcal{E}}}}}}}}$ already excludes, e.g., time-dependent effects in between different experiments, and the factorization into a map *ϕ* further restricts to Markovian noise. Under this assumption, it can be proven that RB protocols^9–14^ function correctly^14,34,35^. For non-Markovian noise much less is known, but in the context of RB rigorous results have been obtained for quasi-static noise^36^, time-dependent noise^37^ and more recently using tensor-models^38^. We expect these results to broadly carry over to random sequence estimation.

Second, we assume gate-independent noise, positing the existence of quantum channels Λ_L_, Λ_R_ such that

3 $$\phi (g)={{{\Lambda }}}_{L}\omega (g){{{\Lambda }}}_{R}$$

where $\omega (g)(\rho )={U}_{g}\rho {U}_{g}^{{{{\dagger}}} }$ is some ideal implementation of the gate *g*. We argue in the section “ Gate-dependent noise” that our results also apply (up to a negligible error) in the more general Markovian error model, but rigorously proving this (along the lines of ref. ^14^) is beyond the scope of this work.

Instead of Λ_R_, Λ_L_ describing the noise of the gate-set implementation, one can also take the perspective of actively interleaving a channel of interest between a fairly ideal implementation of a gate-set (as is done in interleaved randomized benchmarking^30^). While different in protocol and data interpretation, in the analysis, this *black-box query model*

4 $$\phi (g)={{\Lambda }}\omega (g)$$

is simply a special case of the gate-independent noise model and results carry over.

The main analytical result of this work is to establish rigorous performance guarantees for the estimation from gate-set shadows. The obvious first question being: what do we actually estimate? As a first result, we establish the ‘simple’ model that we should be fitting to the data. We show that for a probe operator *A* the empirical estimator of the protocol converges in probability with the number of samples *S* in the shadow to a matrix-exponential decay

5 $${\hat{k}}_{f_A}(m) \quad\mathop{\longrightarrow}\limits^{S \to \infty}\quad k_{f_A}(m)={{{{{\mathrm{Tr}}}}}}[{{\Theta}} {{\Phi}}^{m-1}].$$

Here, the matrix Φ depends only on the ‘between-gates noise channel’ Λ ≔ Λ_R_Λ_L_ and the probe super-operator *A*, while Θ captures SPAM dependence. In particular, if *ω* contains *t* copies of the representation *σ* then we have

6 $${{{\Phi }}}_{i,j}=\frac{1}{|{P}_{j}|}{{{{{{{\rm{Tr}}}}}}}}({P}_{i}A{P}_{j}{{\Lambda }})$$

where *P*_*i*_ is the projector onto the *i*th copy of the representation *σ* inside *ω*. Note that here the trace is taken on the space of super-operators. We give the derivation of this result in Supplementary Note 4, in supplements that cite also refs. ^39–45^.

Equation (5) indicates that we should fit a linear combination of (up-to) *t* exponential decays to the sequence average ${\hat{k}}_{{f}_{A}}(m)$. The resulting decay parameters are the eigenvalues of the matrix Φ, which encode information about the overlap of Λ and *A* in the representation space.

A particularly simple fitting model with easily interpretable decay parameters arises when the representation *σ* appears in the decomposition of *ω* without multiplicities (i.e., there is no other representation in *ω* related to *σ* by a change of basis). If *σ* is multiplicity-free, then ${k}_{{f}_{A}}(m)$ describes a single scalar exponential decay

7 $${k}_{{f}_{A}}(m)\propto {p}_{\sigma,A}{({{\Lambda }})}^{m-1},$$

with decay parameter

8 $${p}_{\sigma,A}({{\Lambda }})=\frac{1}{{d}_{\sigma }}{{{{{{{\rm{Tr}}}}}}}}[{A}_{\sigma }{{\Lambda }}]$$

and *A*_*σ*_ = *P*_*σ*_*AP*_*σ*_ the probe operator restricted to the representation *σ* of dimension *d*_*σ*_. Note that the proportionality now hides the SPAM-dependent pre-factor.

Thus, by fitting a single exponential decay to the empirically observed sequence averages ${\hat{k}}_{{f}_{A}}$, we can estimate *p*_*σ*,*A*_(Λ), the trace-overlap of Λ with *A* on *σ*. The decay parameter can be thought of as a *generalized fidelity* or effective depolarization parameter, indicating how much the noise channel Λ agrees on average with the probe operator *A* on the representation space of *σ*.

### Sample complexity

Against the background of the extensively explored variants of RB protocols, the above decay model is not entirely unexpected. A priori less obvious, however, is the sample efficiency of gate-set shadow protocols. The sequence correlation functions ${f}(x,\, {{{{{\boldsymbol{g}}}}}})$ involve normalization factors that typically scale with the dimension of the irreducible representation under consideration. As a consequence their range can become exponentially large in the number of qubits, causing a simple empirical mean estimator to be susceptible to outliers in the measurement statistics, as well as making a suitably bounded variance a priori nontrivial. Going significantly beyond the established statistical guarantees in RB, we establish general variance bounds for the UIRS protocol. We do this by introducing a sequence analog to the shadow norm introduced in ref. ^16^ defined on probe super-operators *A* as opposed to observables. Emphasizing its explicit dependence on the sequence length *m* we call this norm (really a family of norms indexed by *m*) the *dynamic shadow norm* ∥*A*∥_dyn,*m*_. This norm, formally defined in Supplementary Equation (25), depends on the underlying gate-set ${\mathbb{G}}$ as well as the ideal input POVM {*E*_*x*_} and state *ρ*. Given these parameters, it quantifies the sample complexity of estimating the mean ${k}_{{f}_{A}}(m)$ for arbitrary gate-independent noise. Because of its dependence on the sequence length, the dynamic shadow norm is a more intricate object than its state counterpart. Evaluating it for specific gate sets accounts for the bulk of the technical innovation in this paper. In terms of the dynamic shadow norm we have the following upper bound on the variance of the UIRS protocol.

#### Theorem 1

(Upper bound on the variance). *Consider an UIRS protocol (at sequence length* *m) with gate-set* ${\mathbb{G}}$ *and a correlation function* *f*_*A*_ *with probe operator* *A*. *The variance of the associated mean* ${k}_{{f}_{A}}(m)$ *is bounded as*

9 $${{\mathbb{V}}}_{A}(m)\le \parallel A{\parallel }_{{{{{{{{\rm{dyn}}}}}}}},m}\,.$$

An extended statement and the proof is given in Supplementary Note 4. The bound on the variance ${{\mathbb{V}}}_{A}(m)$ directly implies a non-asymptotic bound on the sample complexity for the estimator ${\hat{k}}_{{f}_{A}}(m)$ with exponential confidence through the use of median-of-means estimation. The exponential confidence in particular allows us to estimate ‘many’ quantities simultaneously from the same shadow data with only logarithmic overhead in the number of quantities. See Supplementary Note 3 for details. More precisely, we get the following guarantee: Run the UIRS protocol (at sequence length *m*) and measure a gate-set shadow of *S* many samples. Choose a set ${{{{{{{\mathcal{A}}}}}}}}$ of probe operators, an *ϵ* > 0 and ensure that for all $A\in {{{{{{{\mathcal{A}}}}}}}}$

10 $$S\ge C\parallel \!A{\parallel }_{{{{{{{{\rm{dyn,m}}}}}}}}}\frac{\log (|{{{{{{{\mathcal{A}}}}}}}}|)}{{\epsilon }^{2}}$$

for a suitable constant *C*. Then, in the post-processing, we obtain *ϵ*-additive estimates, i.e., $|{k}_{A}(m)-{\hat{k}}_{A}(m)|\le \epsilon$ for all $A\in {{{{{{{\mathcal{A}}}}}}}}$.

Hence, bounding the dynamic shadow norm for all $A\in {{{{{{{\mathcal{A}}}}}}}}$ and different sequence lengths *m* gives simultaneous guarantees for many estimators ${\hat{k}}_{A}(m)$ with an overall sampling complexity being the sum of the bounds Equation (10) for all *m*. As explained above, $m \, \mapsto \, {\hat{k}}_{A}(m)$ is then fitted using a theoretical signal model. For example, in the scenario of multiplicity-free representations giving rise to a single exponential decay Eq. (7), we thereby obtain an estimator for *p*_*σ*,*A*_(Λ) for all $A\in {{{{{{{\mathcal{A}}}}}}}}$. The exponential fitting itself is a well-studied problem, for which many advanced techniques^46,47^, flexible software packages^48^, and rigorous bounds^49^ can be readily applied.

### Example: Multi-qubit Clifford UIRS

We now provide two particularly practically relevant examples of UIRS protocols, derive their signal model and a dynamical shadow norm bound guaranteeing their efficiency.

The first example is the multi-qubit Clifford group ${{\mathbb{C}}}_{n}$ that already takes a prominent role in quantum characterization and quantum computation more generally^50^. We consider an UIRS experiment for ${{\mathbb{C}}}_{n}$: i.e., sequences of i.i.d. Clifford gates uniformly drawn at random, acting on the initial state $\left|0\right\rangle \left\langle 0\right|$ and ending in a computational basis measurement. This is a common gate-set with a well-understood representation structure, allowing us to explicitly calculate the sequence mean *k*_*A*_(*m*) and give bounds on the dynamic shadow norm ∥*A*∥_dyn,*m*_ which controls the sample complexity of sequence estimation.

#### Signal model

The adjoint representation of the multi-qubit Clifford group *ω*(*g*) decomposes into two inequivalent irreducible representations^51^: *σ*_*tr*_ supported on the normalized identity matrix and *σ*_ad_ supported on the space of traceless matrices, spanned by the generalized Pauli matrices. See Supplementary Note 2 for details. We focus on sequence correlation functions with support on *σ*_ad_ only, i.e., *A* = *P*_ad_*AP*_ad_. Then, ${k}_{{f}_{A}}(m)$ describes a single exponential decay Eq. (7) with

11 $${p}_{{{{{{{{\rm{ad}}}}}}}},A}({{\Lambda }})=\frac{1}{{2}^{2n}-1}{{{{{{{\rm{Tr}}}}}}}}({P}_{{{{{{{{\rm{ad}}}}}}}}}A{P}_{{{{{{{{\rm{ad}}}}}}}}}{{\Lambda }})\,.$$

This is a familiar quantity: For *A* = *P*_ad_, it corresponds to the depolarizing probability (essentially the average fidelity) of the channel Λ. As a very special case, the Clifford UIRS protocol in this way emulates standard Clifford randomized benchmarking without performing an inversion. However, gate-set shadows are considerably more flexible. For instance, by choosing *A* = *U* a unitary channel, *p*_ad,*U*_(Λ) measures the relative average fidelity of Λ w.r.t. the unitary *U* (i.e., the average fidelity of *U*^†^∘Λ). In particular, for *U* a Clifford channel, the corresponding sequence correlation function can be evaluated efficiently. Relative average gate fidelities are also estimated in interleaved RB. Compared to existing interleaved RB protocols such as the scheme of ref. ^29^, gate-set shadows have the crucial advantage that the experimental protocol itself is independent of *U*.

Since we do not have to implement *A* on a quantum device, we can also consider *A* that do not correspond to quantum channels such as rank-one super-operators of the form $X\,{{{{{{{\rm{Tr}}}}}}}}(Y \, \cdot \, )$ for operators *X*, *Y*. Hence, the gate-set shadows are a versatile tool to estimate properties of the implementation of a Clifford gate-set.

#### Dynamical shadow norm

The versatility of Clifford UIRS in practice of course crucially depends on the sample efficiency of the estimation. From the above, it is not clear that *k*_*A*_(*m*) can be efficiently estimated for arbitrary *A*. Demanding that *k*_*A*_(1) = 1 in the limit of perfect state preparation, measurement, and gates, the normalization factor *α* in Eq. (2) is *α* = 2^*n*^ + 1, leading to a single-shot estimator taking values exponentially large in *n*. Building upon the machinery of the dynamic shadow norm and Theorem 1, we can still provide guarantees for efficiently estimatable probe operators and investigate the limits of Clifford UIRS. As a first step, we assume *A* to be a restriction of a unitary channel *U* to the traceless subspace, i.e., *A* = *P*_ad_*UP*_ad_. In this case, the dynamic shadow norm can in fact be bounded by a small constant independent of the sequence length.

##### Theorem 2

(Clifford UIRS unitary norm bound). *For the* *n*-q*ubit Clifford UIRS protocol*, *U* *a unitary channel, and* *A* = *P*_ad_*UP*_ad_, *it holds that*

12 $$\parallel \!A{\parallel }_{{{{{{{{\rm{dyn}}}}}}}},m}\le 10\,.$$

Theorem 2 is noteworthy for several reasons. First, it does not depend on the number of qubits *n*. Therefore, the estimation of *k*_*U*_(*m*) is efficient even on a quantum system consisting of many qubits. Second, the shadow-norm bound does not depend on the sequence length *m*, enabling relative accuracy estimation of the decay rate in certain regimes. We note that the constant 10 is probably sub-optimal. The derivation of this theorem can be found in Supplementary Note 6.

As the main consequence of Theorem 2 together with Eq. (10), we find that it is possible to sample-efficiently estimate exponentially many relative fidelities with respect to unitary channels to additive precision from the same gate-set shadows obtained by multi-qubit Clifford UIRS.

Next, we consider a general probe super-operator *A* restricted to the traceless subspace. Note that *A* does not need to be a quantum channel. In the following, we show that the dynamical shadow norm can be controlled in terms of the unitarity^52^ of *A*,

13 $$u(A)={{{{{{{\rm{Tr}}}}}}}}(A{A}^{{{{\dagger}}} }){({2}^{2n}-1)}^{-1}\,.$$

For instance, *u*(*A*) ≤ 1 if *A* is a quantum channel with equality if *A* is indeed unitary. We prove the following theorem.

##### Theorem 3

(Clifford UIRS general norm bound). *Consider the* *n*-*qubit Clifford UIRS protocol and let* *A* = *P*_ad_*AP*_ad_ *be a probe super-operator restricted to the traceless subspace. The dynamic shadow norm for* *m* > 2 *is upper bounded by*

14 $$\parallel \!A{\parallel }_{{{{{{{{\rm{dyn}}}}}}}},m}\le C\,{m}^{2}r{(A)}^{m-1}\max \{r(A),\, 1\},$$

*with* *r*(*A*) = (1 + 2^4−*n*/3^)*u*(*A*) * and suitable constant C*.

The proof of this theorem, given in Supplementary Note 6, is similar in spirit to Theorem 2, but significantly more involved. Choosing *A* to be unitary (*u*(*A*) = 1) does not recover Theorem 2, due to the appearance of the quadratic scaling in *m*. This term arises because we consider general probe super-operators *A*, giving rise to polynomial transient dynamics in the dynamic shadow norm (due to the non-normality of the underlying operators [ref. ^53^, Chapter 6]). For many sensible choices of *A*, the polynomial scaling in *m* does not appear as is evidenced by theorem 2. Also, the bound does not quite scale with the unitarity *u*(*A*), but rather with the parameter *r*(*A*) which differs from *u*(*A*) by an exponentially small factor. We believe this to be an artifact of the proof technique.

This theorem leads us to the remarkable conclusion that the multi-qubit Clifford UIRS protocol allows us to estimate overlaps *p*(*A*Λ) for a very large class of super-operators. In particular, *A* can be any trace non-increasing map, allowing us, e.g., to characterize the overlap between the noise channel Λ and sets of Kraus operators, making the Clifford UIRS protocol an all-purpose tool for noise map exploration.

### Example: local Clifford UIRS

A particularly scalable and interesting protocol arises when performing a UIRS protocol with the local Clifford group ${{\mathbb{C}}}_{1}^{\times n}$ over *n* qubits. In this case, the experiment consists of performing sequences of i.i.d. random single-qubit gates simultaneously on all qubits, initially prepared in $\left|0\right\rangle \left\langle 0\right|$ ending with a computational basis measurement.

For ${{\mathbb{C}}}_{1}^{\times n}$ the conjugate representation $\omega (g)={U}_{g}\cdot {U}_{g}^{{{{\dagger}}} }$ with ${U}_{g}={U}_{({g}_{1},\ldots,{g}_{n})}={U}_{{g}_{1}}\otimes \ldots \otimes {U}_{{g}_{n}}$ decomposes into 2^*n*^ irreducible, mutually inequivalent representations *σ*_*w*_ with *w* ∈ {0, 1}^*n*^ that have support on the normalized non-identity Pauli operators on all qubits *i* for which *w*_*i*_ = 1. We denote the projectors onto these irreducible sub-representations as *P*_*w*_ (see Supplementary Note 2 for more details).

#### Signal model

We consider sequence correlation functions with probe operator *A* that only have support on a single irreducible representation *σ*_*w*_(*g*) and set *α* = 2^*n*^3^∣*w*∣^. Then, the mean ${k}_{{f}_{A}}(m)$ again describes a single exponential decay Eq. (7) with

15 $${p}_{w,A}({{\Lambda }})={{{{{{{\rm{Tr}}}}}}}}({P}_{w}{{\Lambda }}{P}_{w}A){3}^{-|w|}\,.$$

We will refer to this quantity as a *local fidelity w.r.t. A*. The local fidelity is again somewhat familiar. The special case ${p}_{w,{\mathbb{I}}}$ has been called the ‘addressability’ in ref. ^28^, where it was used to gain information about the strength of correlated errors. Using gate-set shadows of simultaneously applied local gate sequences, we can collect even more information about correlated errors, giving rise to an efficient *cross-talk tomography protocol* introduced in the section “Application: Cross-talk tomography”. We can again equip the UIRS protocol with sampling complexity guarantees by bounding the shadow norm.

#### Dynamic shadow norm

We derive a bound on the dynamic shadow norm of the local Clifford group that depends exponentially on the Hamming weight ∣*w*∣ of the bit-string *w* labeling the representation being addressed but is independent of the total number of qubits in the system.

##### Theorem 4

(Local Clifford UIRS norm bound). *For the local Clifford UIRS protocol on* *n* *qubits*, *w* ∈ {0, 1}^*n*^, *and* *A* = *P*_*w*_*AP*_*w*_ *a probe operator, it holds that*

16 $$\parallel \!A{\parallel }_{{{{{{{{\rm{dyn}}}}}}}},m}\le {2}^{|w|}{3}^{2|w|}{\left[{3}^{-|w|}{{{{{{{\rm{Tr}}}}}}}}(A{A}^{{{{\dagger}}} })\right]}^{m-1}\,.$$

The proof is given in Supplementary Note 5. Note that the term inside the square bracket in Eq. (16) can be considered as a variant of the unitarity restricted to the image of *P*_*w*_. In particular, if *A* = *P*_*w*_*UP*_*w*_ for any unitary channel *U* we have ${3}^{-|w|}{{{{{{{\rm{Tr}}}}}}}}(A{A}^{{{{\dagger}}} })=1$. Thus, for restrictions of unitary probe operators, the bound becomes independent of the sequence length and in consequence, the protocol is sample-efficient for bounded ∣*w*∣.

### Example beyond UIRS: Pauli-noise estimation

Thus far we have focused on uniformly independently sampled random sequences (UIRS protocols). It is also fruitful to consider more general probability distributions on the set of sequences of a given length. We give an example of this by constructing a simple protocol that estimates the diagonal elements of an *n*-qubit channel Λ using only *O*(*n*2^*n*^) samples. This sampling complexity matches the asymptotic bound given for this task in ref. ^19^. Using gate-set shadows, however, gives a simpler experimental description and analysis. To this end, consider random sequences of the form ${{{{{\boldsymbol{g}}}}}}=({c}^{-1},\, {p}_{m},\, \ldots,\, {p}_{1},\, c)$ where *p*_1_, …, *p*_*m*_ are chosen independently uniformly at random from the Pauli group ${{\mathbb{P}}}_{n}$ and *c* is chosen uniformly at random from the Clifford group ${{\mathbb{C}}}_{n}$. Note the inverse *c*^−1^ here at the end of the sequence. In a black-box fashion, we additionally intersperse the channel Λ in between executing the random Pauli elements in the experiment. The measurement is again a computational basis measurement and the initial state $\rho=\left|0\right\rangle \left\langle 0\right|$. Choose *τ* to be a Hilbert–Schmidt normalized traceless Pauli operator. As the associated correlation function, we define

17 $${f}_{\tau }(x,\, {{{{{\boldsymbol{g}}}}}}): \!=\alpha \,{{{{{{{\rm{Tr}}}}}}}}[{E}_{x} \omega (c) \omega ({p}_{m}){A}_{\tau }\ldots {A}_{\tau } \omega ({p}_{1}) \omega (c) \rho ]$$

with ${A}_{\tau }: \!=\tau \, {{{{{{{\rm{Tr}}}}}}}}(\tau \,\cdot )$ and *α* = 2^*n*^(2^*n*^ + 1). For convenience, we ignore the SPAM in deriving and stating the following results. Both of these assumptions can be easily relaxed. As we show in Supplementary Note 7, the corresponding sequence mean is the power of the diagonal matrix entry of Λ corresponding to *τ*, i.e.,

18 $${k}_{\tau }(m)={{{{{{{\rm{Tr}}}}}}}}{[\tau {{\Lambda }}(\tau )]}^{m-1}.$$

We further show that the variance of the associated estimator can be bounded as

19 $${{\mathbb{V}}}_{\tau }(m)\le \frac{{2}^{3n}{({2}^{n}+1)}^{3}}{{2}^{3n}({2}^{2n}-1)}=O({2}^{n}),$$

for all choices of *τ*. Note that there are 4^*n*^−1 such choices, characterizing all diagonal elements of the quantum channel Λ. Hence, by using median-of-means estimators, we can estimate *k*_*τ*_(*m*) for all *τ* to uniform additive precision using *O*(*n*2^*n*^) samples (independently of *m*). By the analysis in ref. ^49^ for the estimation of single exponential decays and the fact that the decay rates Λ_*τ*,*τ*_ are strongly clustered (ref. ^33^, Lemma 4) leads to a relative precision estimation of the associated Pauli fidelities, matching the performance given in ref. ^19^.

### Application: Learning unitary noise models

In the previous section, we have shown how to efficiently estimate the overlap of certain probe operators with the noise of a gate-set. This data, e.g., the average gate fidelity of the noise with a specific gate, is already of interest. The most intriguing feature, however, is that we can estimate many different probe operators from the same data. In this way, we can use estimates from gate-set shadows as a subroutine in a complex post-processing pipeline that extracts more information about the noise. This opens up the way to perform many different characterization tasks that arise in a full-scale engineering cycle of building a quantum computer from the same simple data. Importantly, the resulting protocols automatically inherit the SPAM robustness of the estimation protocol. We illustrate these possibilities with three concrete examples.

**Fig. 2:**
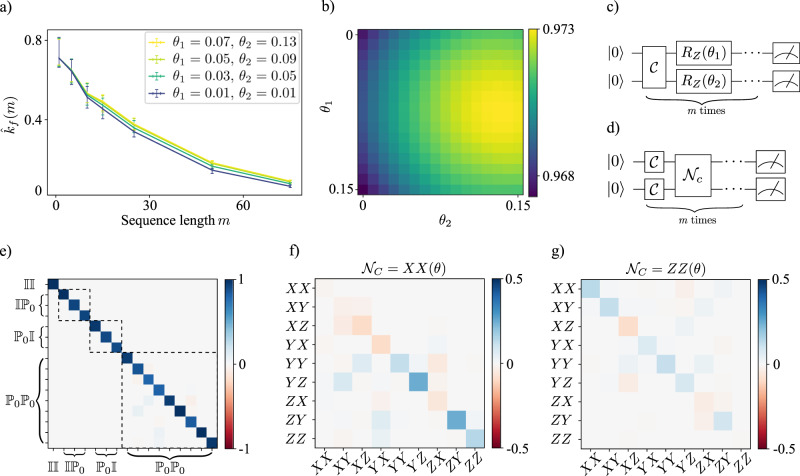
Numerical simulations of two potential applications, unitary noise optimization (section “Application: Learning unitary noise models”) and cross-talk tomography (section “Application: Cross-talk tomography”). Panels **a** and **b** show simulation results of the multi-qubit Clifford UIRS protocol for two qubits and 1000 random sequences per sequence length. Between every Clifford gate ${{{{{{{\mathcal{C}}}}}}}}$, two independent *Z*-rotations *R*_*Z*_(*θ*) with rotation angles *θ*_1_ = 0.07 and *θ*_2_ = 0.13 have been applied (see circuit diagram **c**). Panel **b** shows average fidelities *F*(*U*(*θ*), Λ) reconstructed from the gate-set shadows using the ansatz *U*(*θ*_1_, *θ*_2_) = *R*_*Z*_(*θ*_1_) ⊗ *R*_*Z*_(*θ*_2_). Example decays of the sequence averages *k*(*m*) are shown in panel **a** with bootstrapped 95% confidence intervals around the decay points. Panels **e**–**g** display simulation results for cross-talk tomography from two-qubit local Clifford UIRS data with 15,000 random sequences per sequence length. After every layer of local Cliffords, an entangling cross-talk noise process ${{{{{{{{\mathcal{N}}}}}}}}}_{c}$ has been applied (see the circuit diagram (**d**). Panel **e** shows the *Pauli transfer matrix* (PTM) of the reconstructed pinched marginal *S* given in Supplementary Equation (107), for cross-talk of the form ${{{{{{{{\mathcal{N}}}}}}}}}_{c}=XX(\theta )$, with dashed boxes indicating the unital marginals Λ_0,1_, Λ_1,0_, and Λ_1,1_. Panels **f** and **g** show the PTMs of the difference between the unital marginal Λ_1,1_ and the tensor product Λ_1,0_ ⊗ Λ_0,1_ as a characterization of the cross-talk between the two qubits, for cross-talk ${{{{{{{{\mathcal{N}}}}}}}}}_{c}=XX(\theta=0.4)$ in **f** and ${{{{{{{{\mathcal{N}}}}}}}}}_{c}=ZZ(\theta=0.4)$ in (**f**). Simulations have been performed using Qiskit^69^ with single-qubit depolarizing noise of *p*_1_ = 0.002 for single-qubit gates and two-qubit depolarizing noise *p*_2_ = 0.01 for two-qubit gates (on top of the custom noise processes after each Clifford layer). For the PTM plots, modified functions from the Forest Benchmarking package^70^ have been used.

When characterizing noisy quantum gates one differentiates between coherent noise (due to imperfect specification of the gate) and incoherent noise (due to interactions with the environment). These two types of noise have different consequences, for e.g., error correction^1,54^ and are engineered away in different ways. At the same time, coherent errors can be corrected by experimental design and control if one has a concrete description. Given a model for a unitary channel *θ* ↦ *U*(*θ*), we can learn the model parameters *θ* approximating the noise channel Λ by maximizing *F*(*U*(*θ*), Λ). During the optimization, the objective function, its gradient, etc. can be estimated from the same classical gate-set shadow. For the multi-qubit Clifford UIRS, every estimation requires a polynomial-size shadow in the number of qubits and only a logarithmic overhead in the number of evaluations *F*(*U*(*θ*), Λ). A numerical simulation of a simple learning example is given in Fig. 2.

### Application: Cross-talk tomography

A key source of error in today’s quantum computing devices is correlated noise or cross-talk. For this reason, a significant effort has gone into characterizing cross-talk errors specifically^28,55,56^. Using the flexibility of extracting manifold information from gate-set shadows in the post-processing, we here propose *cross-talk tomography* as an efficient, robust, and detailed cross-talk characterization procedure, based on the local Clifford UIRS protocol.

The protocol gains tomographic information about, what we call, the *unital marginals* Λ_*w*_ = *P*_*w*_Λ*P*_*w*_, *w* ∈ {0, 1}^*n*^, of the noise channel Λ. (Here, *P*_*w*_ is again the projector onto the irreducible representations of the local Clifford group.) These unital marginals arise as restrictions of channel marginals ${{{\Lambda }}}_{\bar{A}}$, where one evaluates Λ on a maximally mixed input on a system *A* and traces out the resulting state on *A*^57^.

Now Λ_*w*_ can be reconstructed via simple linear inversion (see ref. ^32^, Lemma 37) from the local fidelities ${p}_{w,{{{{{{{\mathcal{C}}}}}}}}}({{\Lambda }})={3}^{-|w|}{{{{{{{\rm{Tr}}}}}}}}({{\Lambda }}{P}_{w}{{{{{{{\mathcal{C}}}}}}}}{P}_{w})$ with respect to the probe-operators given by the local Clifford channel ${{{{{{{\mathcal{C}}}}}}}}$ according to

20 $${{{\Lambda }}}_{w}=\frac{1}{|{{\mathbb{C}}}_{w}|}\mathop{\sum}\limits_{{{{{{{{\mathcal{C}}}}}}}}\in {{\mathbb{C}}}_{w}}{3}^{2|w|}{p}_{w,{{{{{{{\mathcal{C}}}}}}}}}({{\Lambda }}){{{{{{{{\mathcal{C}}}}}}}}}^{{{{\dagger}}} }{P}_{w}\,,$$

where the sum is restricted to local Clifford channels with unitaries from the subgroup ${{\mathbb{C}}}_{w}$ of ${{\mathbb{C}}}_{n}$ acting non-trivially on only the qubits in the support of *w*. In fact, it is sufficient to consider all local Clifford channels ${{{{{{{\mathcal{C}}}}}}}}$ that act non-trivially on the support of the bit-string *w*. Not restricting the non-trivial support of ${{{{{{{\mathcal{C}}}}}}}}$, however, allows us to simultaneously reconstruct Λ_*w*_ for multiple values of *w*.

This constitutes the basis of cross-talk tomography for *k*-local interactions. Let *H*_*k*_ ⊂ {0, 1}^*n*^ be the subset of bit strings with Hamming weight *k*. (i) Perform the UIRS experiment for the local Clifford group over *n* qubits. (ii) Estimate ${p}_{w}({{{{{{{\mathcal{C}}}}}}}})$ for all *w* ∈ *H*_*k*_ and for all ${{{{{{{\mathcal{C}}}}}}}}$ acting non-trivially on the support of *w*. (iii) Reconstruct all Λ_*w*_ for *w* ∈ *H*_*k*_.

By comparing Λ_*w*_ for different bit strings, one obtains information about the correlations present in Λ. Building upon the guarantees for UIRS, we show that cross-talk tomography is *ϵ*-accurate in the diamond norm for all Λ_*w*_ using *O*(*k*^2^ 2^9*k*^/*ϵ*^2^) shadow samples (up-to log-factors). Thus, for small *k*, cross-talk tomography is highly scalable to large numbers of qubits. In light of Theorem 4, this efficiency stems from using local unitary probe operators. The derivation and even tighter guarantees are given in Supplementary Note 8.

As an illustration, we study the protocol with a 2-qubit example. We start by using the local Clifford UIRS protocol to reconstruct the 2-qubit unital marginals Λ_1,0_, Λ_0,1_ and Λ_1,1_. Next, we compute the tensor product Λ_1,0_ ⊗ Λ_01_. It is straightforward to see that if the channel Λ is a tensor product of single-qubit quantum channels featuring no correlations (i.e., there is no cross-talk) then Λ_1,0_ ⊗ Λ_0,1_ = Λ_1,1_. Hence, both the difference Λ_1,0_ ⊗ Λ_0,1_−Λ_1,1_ and the product ${{{\Lambda }}}_{1,1}{({{{\Lambda }}}_{1,0}\otimes {{{\Lambda }}}_{0,1})}^{-1}$ provide meaningful characterizations of cross-talk present between qubits 1 and 2. The difference measure can be considered as a generalization of the commonly used addressability metric proposed in ref. ^28^. But going beyond a mere metric, we expect that the channel marginals not only detect the presence of cross-talk but also provide more detailed diagnostic information. As a proof of principle, we have numerically simulated the above protocol to diagnose cross-talk in a two-qubit system. The results of a numerical simulation of the protocol are presented in Fig 2.

### Application: SPAM-robust channel reconstruction

Kimmel et al.^29^ have proposed the idea to combine the output of *O*(2^4*n*^) different interleaved RB experiments in order to get a robust tomographic estimate of an unital quantum channel Λ. By explicitly exploiting the low Kraus-rank, *compressive RB tomography*^31,32^ can reconstruct a unitary approximation to the quantum channel from (up-to-log-factors) *O*(2^2*n*^) randomly selected different relative average-gate fidelities with respect to Clifford unitaries. The previous references, however, left the problem open of providing a SPAM-robust RB protocol that achieves the information-theoretically optimal sampling complexity of *O*(2^4*n*^)^32^ for reconstructing a unitary channel.

We fill in this blank using the data from a multi-qubit Clifford UIRS protocol. Using a set of randomly selected Clifford unitaries as probe operators, we can provide the input data to the reconstruction algorithm of ref. ^32^. We show in Supplementary Note 9 that the number of gate-set shadows to guarantee an accurate reconstruction (in Hilbert-Schmidt norm of the Choi-states) indeed matches the lower bound of *O*(2^4*n*^). Note that the number of channel invocations is bounded by the maximal sequence length times the number of sequences. Besides the favorable scaling, the UIRS protocol has a crucial advantage compared to, e.g., the interleaved protocol of ref. ^29^ that the same measurement data is used for estimating all the average fidelities.

Going beyond the compressive reconstruction of unitary quantum channels, we can use Clifford UIRS as a primitive for the robust reconstruction of arbitrary unital quantum channels in the spirit of ref. ^29^, see also ref. [^32^, Theorem 38] and ref. ^58^. The required size of the gate-set shadow is *O*(2^8*n*^) for accurate reconstruction in any norm in which unitary channels are normalized.

### Gate-dependent noise

The presentation so far assumed gate-independent noise. This assumption can be substantially relaxed, at the cost of introducing a more complex description of the noise. We will focus on the UIRS protocol, which is particularly robust against gate-dependent fluctuations. We give a fairly comprehensive argument but leave rigorous proof of the robustness to future work. Our argument follows that of the robustness against gate-dependent errors for RB^14,34^. For gate-dependent noise, the data form in expectation can be generally written as

21 $${k}_{A}(m)={{{{{{{\rm{Tr}}}}}}}}\left[{{\Xi }}\,(A\otimes {\mathbb{I}}){({{{{{{{\mathcal{F}}}}}}}}(\phi )[\sigma ])}^{m-1}\right],$$

where Ξ depends on the state and measurement and the operator ${{{{{{{\mathcal{F}}}}}}}}(\phi )[\sigma ]: \!\!={{\mathbb{E}}}_{g\in {\mathbb{G}}}\,\sigma (g)\otimes \phi (g)$ is known as the (non-commutative) Fourier transform of *ϕ*, evaluated at the irreducible representation *σ*; see the derivation of Theorem 7 in  Supplementary Information.

A key fact about this Fourier transform (see, e.g., ref. ^59^ for proof) is that if *ϕ* is a representation *ω* (i.e., a perfectly implemented gate-set), then ${{{{{{{\mathcal{F}}}}}}}}(\phi )[\sigma ]$ is an orthogonal projector with rank equal to the number of copies of *σ* present in *ω*. For simplicity, let *ω* be multiplicity-free. Then, ${{{{{{{\mathcal{F}}}}}}}}(\phi )[\sigma ]$ is a rank-one projector. This implies that $(A\otimes {\mathbb{I}}){{{{{{{\mathcal{F}}}}}}}}(\phi )[\sigma ]$ is also a rank-one projector. When *ϕ* is a sufficiently ‘good’ implementation of *ω*, the difference between ${{{{{{{\mathcal{F}}}}}}}}(\phi )[\sigma ]$ and ${{{{{{{\mathcal{F}}}}}}}}(\omega )[\sigma ]$ is small (in some suitable norm) and can be regarded as a perturbation of ${{{{{{{\mathcal{F}}}}}}}}(\omega )[\sigma ]$. (See ref. ^14^ for a discussion of norms on this space.) Applying the perturbation theory of non-normal matrices, we conclude that $(A\otimes {\mathbb{I}}){{{{{{{\mathcal{F}}}}}}}}(\phi )[\sigma ]$ is as well approximately rank-one, and in particular that there exist super-operators Λ_*L*_, Λ_*R*_ such that

22 $${\left((A\otimes {\mathbb{I}}){{{{{{{\mathcal{F}}}}}}}}(\phi )[\sigma ]\right)}^{m}={\left(A{{{\Lambda }}}_{R}{P}_{\sigma }{{{{{{{\rm{Tr}}}}}}}}[{P}_{\sigma }{{{\Lambda }}}_{L}\cdot ]\right)}^{m}+{E}^{m}$$

where *E* is a matrix of the small norm and *P*_*σ*_ is the projector onto *σ* (in the image of *ω*). This means that the decay rate *k*_*A*_(*m*) has the general functional form

23 $${k}_{A}(m)={B}_{1}p{(A)}^{m-1}+{B}_{2}\delta {(E)}^{m-1}$$

where *B*_1_, *B*_2_ are real numbers encoding SPAM, *δ*(*E*) is small, and *p*(*A*), the dominant eigenvalue of $(A\otimes {\mathbb{I}}){{{{{{{\mathcal{F}}}}}}}}(\phi )[\sigma ]$, is given by

24 $$p(A)=|{P}_{\sigma }{|}^{-1}{{{{{{{\rm{Tr}}}}}}}}({{{\Lambda }}}_{L}{P}_{\sigma }A{P}_{\sigma }{{{\Lambda }}}_{R})\,.$$

Up to a small and exponentially decreasing error, we thus recover the functional form of Eq. (5) also in the presence of gate-dependent noise. It is important to note, however, that in this general case, Λ_L_ and Λ_R_ (and their product) need not be CPTP. This complicates the interpretation of *p*(*A*) as describing an aspect of a physical noise process.

## Discussion

It has long been known that classical randomness can facilitate the construction of informative characterization protocols for quantum devices. Randomized benchmarking^9–14^ and classical shadow estimation^16,17^ are examples of this mindset. In our work, we follow this paradigm even more stringently for diagnosing noise in gate-set implementations. Instead of engineering sophisticated and specific experimental protocols for a specific task, we turn the approach upside down: we focus on the ‘simplest’ randomized protocol that can be implemented with current and near-term quantum architectures: Random gate sequences followed by native measurements. Accepting this restriction, we then ask how detailed diagnostic information can be extracted from the resulting data and most importantly how many samples are required.

It turns out that the resulting prescription—a single experiment that can and has been implemented experimentally already—allows for solving many benchmarking, certification, and identification problems with (near-)optimal efficiency. All the technicalities that come along with different tasks are shifted to the classical post-processing phase. Most importantly, multiple diagnostic tasks can be performed from the same measurements, allowing us to base an entire engineering cycle on a single experiment.

The ideas advocated here constitute the beginning rather than the conclusion of a program. We regard our theoretical results as a strong motivation to experimentally realize and make use of concrete applications, such as robust learning of unitary noise and cross-talk tomography. In addition, several further extensions seem exciting. A logical first extension of our work is UIRS with other groups and non-uniform measures over said groups. As with state shadow tomography and randomized benchmarking, we believe the UIRS protocol can be furnished with rigorous guarantees for several other useful gate sets such as the matchgates^60,61^, the Heisenberg-Weyl group, the CNOT-dihedral group, and even gate sets that do not constitute a group^62,63^.

We also illustrated the potential of using correlated sequences where the gates are not drawn independently. We believe that using simple correlated sequences gives a fruitful perspective on long-standing problems such as the characterization of non-Markovian and time-varying noise processes in an experimentally friendly and scalable way. Furthermore, while not demonstrated here, akin to their state analog, gate-set shadows can also be used for estimating non-linear quantities.

While the bulk of this work discusses diagnostic tools for developing near-term quantum computing devices, random sequence protocols apply beyond that. We expect that gate-set shadows will for instance find application as a primitive in quantum machine learning^64^, in particular in dynamic settings such as time-series estimation. Also in this context, the possibility to ‘measure first and ask later’ increases the flexibility in devising hybrid quantum-classical schemes with experimentally feasible quantum computations.

### Supplementary information

Supplementary Information

## Data Availability

The simulated data used for creating the plots in Fig. 2 have been deposited on Figshare and are publicly available^68^.

## Code availability

The code used to simulate the protocol and create the plots in Fig. 2 is available upon request.

## References

[1] Campbell ET, Terhal BM, Vuillot C (2017). Roads towards fault-tolerant universal quantum computation. Nature.

[2] Barends R (2014). Superconducting quantum circuits at the surface code threshold for fault tolerance. Nature.

[3] Arute F (2019). Quantum supremacy using a programmable superconducting processor. Nature.

[4] Barak, B., Chou, C.-N. & Gao, X. Spoofing linear cross-entropy benchmarking in shallow quantum circuits. arXiv:2005.02421 (2020).

[5] Hangleiter, D. & Eisert, J. Computational advantage of quantum random sampling. *Rev. Mod. Phys.* **95**, 035001 (2023).

[6] Eisert J (2020). Quantum certification and benchmarking. Nat. Rev. Phys..

[7] Kliesch M, Roth I (2021). Theory of quantum system certification. PRX Quantum.

[8] Elben A (2023). The randomized measurement toolbox. Nat. Rev. Phys..

[9] Knill E (2008). Randomized benchmarking of quantum gates. Phys. Rev. A.

[10] Dankert C, Cleve R, Emerson J, Livine E (2009). Exact and approximate unitary 2-designs and their application to fidelity estimation. Phys. Rev. A.

[11] Emerson J, Alicki R, Zyczkowski K (2005). Scalable noise estimation with random unitary operators. J. Opt. B.

[12] Lévi B, López CC, Emerson J, Cory DG (2007). Efficient error characterization in quantum information processing. Phys. Rev. A.

[13] Magesan E, Gambetta JM, Emerson J (2012). Characterizing quantum gates via randomized benchmarking. Phys. Rev. Lett..

[14] Helsen, J., Roth, I., Onorati, E., Werner, A. H. & Eisert, J. General framework for randomized benchmarking. *PRX Quantum*, **3**, 020357 (2022).

[15] Flammia, S. T. Averaged circuit eigenvalue sampling. arXiv:2108.05803 (2021).

[16] Huang H-Y, Kueng R, Preskill J (2020). Predicting many properties of a quantum system from very few measurements. Nat. Phys..

[17] Paini, M. & Kalev, A. An approximate description of quantum states. arXiv:1910.10543 (2019).

[18] Flammia ST, Liu Y-K (2011). Direct fidelity estimation from few Pauli measurements. Phys. Rev. Lett..

[19] Flammia ST, Wallman JJ (2020). Efficient estimation of Pauli channels. ACM Trans. Quantum Comput..

[20] Merkel ST (2013). Self-consistent quantum process tomography. Phys. Rev. A.

[21] Blume-Kohout, R. et al. Robust, self-consistent, closed-form tomography of quantum logic gates on a trapped ion qubit. arXiv:1310.4492 (2013).

[22] Blume-Kohout R (2017). Demonstration of qubit operations below a rigorous fault tolerance threshold with gate set tomography. Nat. Commun..

[23] Greenbaum, D. Introduction to quantum gate set tomography. arXiv:1509.02921 (2015).

[24] Nielsen E (2020). Probing quantum processor performance with pyGSTi. Quantum Sci. Technol..

[25] Nielsen, E. et al. Gate set tomography. *Quantum*, **5**, 557 (2021).

[26] Gu Y, Mishra R, Englert B-G, Ng HK (2021). Randomized linear gate-set tomography. PRX Quantum.

[27] Brieger R, Roth I, Kliesch M (2023). Compressive gate set tomography. PRX Quantum.

[28] Gambetta JM (2012). Characterization of addressability by simultaneous randomized benchmarking. Phys. Rev. Lett..

[29] Kimmel S, da Silva MP, Ryan CA, Johnson BR, Ohki T (2014). Robust extraction of tomographic information via randomized benchmarking. Phys. Rev. X.

[30] Magesan E (2012). Efficient measurement of quantum gate error by interleaved randomized benchmarking. Phys. Rev. Lett..

[31] Kimmel, S. & Liu, Y. K. Phase retrieval using unitary 2-designs. In *2017 International Conference on Sampling Theory and Applications (SampTA)* pp. 345–349 (IEEE, 2017).

[32] Roth I (2018). Recovering quantum gates from few average gate fidelities. Phys. Rev. Lett..

[33] Helsen J, Wallman JJ, Flammia ST, Wehner S (2019). Multiqubit randomized benchmarking using few samples. Phys. Rev. A.

[34] Wallman JJ (2018). Randomized benchmarking with gate-dependent noise. Quantum.

[35] Proctor T, Rudinger K, Young K, Sarovar M, Blume-Kohout R (2017). What randomized benchmarking actually measures. Phys. Rev. Lett..

[36] Fong, B. H. & Merkel, S. T. Randomized benchmarking, correlated noise, and Ising models. arXiv:1703.09747 (2017).

[37] Wallman JJ, Flammia ST (2014). Randomized benchmarking with confidence. New. J. Phys..

[38] Figueroa-Romero, P., Modi, K. & Hsieh, M. H. Towards a general framework of Randomized Benchmarking incorporating non-Markovian Noise. *Quantum* **6**, 868 (2022).

[39] Fulton, W. & Harris, J. *Representation Theory: a First Course* Vol. 129 (Springer Science & Business Media, 2013).

[40] Devroye, L., Lerasle, M., Lugosi, G. & Oliveira, R. I. Sub-Gaussian mean estimators. *Ann. Statist.* **44**, 2695–2725 (2016).

[41] Nemirowski, A. S. & Yudin, D. B. *Problem Complexity and Method Efficiency in Optimization* (John Wiley and Sons, 1983).

[42] Lugosi G, Mendelson S (2019). Mean estimation and regression under heavy-tailed distributions: a survey. Found. Comput. Math..

[43] Zhu H (2017). Multiqubit Clifford groups are unitary 3-designs. Phys. Rev. A.

[44] Bhatia, R., *Matrix Analysis* Vol. 169 (Springer Science & Business Media, 2013).

[45] Morris, J. & Dakić, B. Selective quantum state tomography. arXiv:1909.05880 (2019).

[46] Roy R, Kailath T (1989). Esprit-estimation of signal parameters via rotational invariance techniques. IEEE Trans. Acoust. Speech Signal Process..

[47] Schmidt R (1986). Multiple emitter location and signal parameter estimation. IEEE Trans. Antennas Propag..

[48] Virtanen P (2020). SciPy 1.0: fundamental algorithms for scientific computing in Python. Nat. Methods.

[49] Harper R, Hincks I, Ferrie C, Flammia ST, Wallman JJ (2019). Statistical analysis of randomized benchmarking. Phys. Rev. A.

[50] Gottesman, D. An introduction to quantum error correction and fault-tolerant quantum computation. In *Quantum information science and its contributions to mathematics, Proceedings of Symposia in Applied Mathematics* (Vol. 68, pp. 13–58) (2010).

[51] Gross D, Audenaert KMR, Eisert J (2007). Evenly distributed unitaries: on the structure of unitary designs. J. Math. Phys..

[52] Wallman J, Granade C, Harper R, Flammia ST (2015). Estimating the coherence of noise. New J. Phys..

[53] Wolf, M. M. *Quantum Channels & Operations: Guided Tour*. *Lecture Notes* Vol. 5 http://www-m5.ma.tum.de/foswiki/pubM (2012).

[54] Huang E, Doherty AC, Flammia S (2019). Performance of quantum error correction with coherent errors. Phys. Rev. A.

[55] Rudinger K (2021). Experimental characterization of crosstalk errors with simultaneous gate set tomography. PRX Quantum.

[56] Maciejewski FB, Baccari F, Zimborás Z, Oszmaniec M (2021). Modeling and mitigation of cross-talk effects in readout noise with applications to the quantum approximate optimization algorithm. Quantum.

[57] Hsieh C-Y, Lostaglio M, Acin A (2022). Quantum channel marginal problem. Phys. Rev. Res..

[58] Scott AJ (2008). Optimizing quantum process tomography with unitary 2-designs. J. Phys. A.

[59] Merkel, S. T., Pritchett, E. J. & Fong, B. H. Randomized benchmarking as convolution: Fourier analysis of gate dependent errors. *Quantum* **5**, 581 (2021).

[60] Helsen J, Nezami S, Reagor M, Walter M (2022). Matchgate benchmarking: scalable benchmarking of a continuous family of many-qubit gates. Quantum.

[61] Zhao A, Rubin NC, Miyake A (2021). Fermionic partial tomography via classical shadows. Phys. Rev. Lett..

[62] Proctor TJ (2019). Direct randomized benchmarking for multiqubit devices. Phys. Rev. Lett..

[63] Liu, Y., Otten, M., Bassirianjahromi, R., Jiang, L., and Fefferman, B., Benchmarking near-term quantum computers via random circuit sampling. arXiv:2105.05232 (2021).

[64] Huang H-Y, Kueng R, Torlai G, Albert VV, Preskill J (2022). Provably efficient machine learning for quantum many-body problems. Science.

[65] Kunjummen, J., Tran, M. C., Carney, D. & Taylor, J. M. Shadow process tomography of quantum channels. *Physical Review A*, **107**, 042403 (2023).

[66] Levy, R., Luo, D. & Clark, B. K. Classical shadows for quantum process tomography on near-term quantum computers. arXiv:2110.02965 (2021).

[67] Chen S, Yu W, Zeng P, Flammia ST (2021). Robust shadow estimation. PRX Quantum.

[68] Helsen, J. et al. Numerical simulations for "Shadow estimation of gate-set properties from random sequences". figshare. Collection. 10.6084/m9.figshare.c.6642551.v2 (2023). 10.1038/s41467-023-39382-9 PMC10439944 37598209

[69] Anis, M. D. S. et al. Qiskit: an open-source framework for quantum computing 10.5281/zenodo.2573505 (2021).

[70] Gulshen, K. et al. Forest Benchmarking: QCVV using PyQuil 10.5281/zenodo.3455847 (2019).

