# Supplementary material for: Shadow estimation of gate-set properties from random sequences
Source: Nat Commun. 2023 Aug 19;14:5039. doi: 10.1038/s41467-023-39382-9 (PMC10439944; doi:10.1038/s41467-023-39382-9)
Supplement: Supplementary file 1 — Supplementary Information [file 41467_2023_39382_MOESM1_ESM.pdf]

Supplementary information: Shadow estimation of gate-set properties from random sequences

J. Helsen, M. Ioannou, J. Kitzinger, E. Onorati, A. H. Werner, J. Eisert, and I. Roth

SUPPLEMENTARY NOTE 1. – NOTATION

Throughout this work, we are operating in the *Liouville* or *transfer matrix representation* of quantum channels. We represent finite-dimensional $d \times d$ density matrices ρ as length d^2 column vectors $|\rho\rangle\rangle$ and POVM elements E as length d^2 row vectors $\langle\langle E|$, with a corresponding trace-inner product $\langle\langle E|\rho\rangle\rangle = \mathrm{Tr}(E^\dagger \rho)$. In this picture, super-operators $\mathcal{E}$ get mapped to $d^2 \times d^2$ matrices $\mathcal{E}$ with the property $\mathcal{E}|\rho\rangle\rangle = |\mathcal{E}(\rho)\rangle\rangle$. Note that this representation is compatible with composition of super-operators (mapping to matrix multiplication), and the taking of tensor products. When d is a power of two a good basis for the space of matrices is the set of Hermitian Pauli operators $\mathbb{P}^*$ (normalized under the trace inner product), in this case we also always write $d = 2^n$. We denote the normalized identity by $2^{-n/2}\mathbb{I} = \tau_0$ and the set of normalized traceless Hermitian Pauli matrices τ as $\mathbb{P}^*_0$. Finally, we use a tilde to indicate noisy implementations of POVM elements and states, so $\tilde{\rho}$ is a noisy implementation of the state ρ and $\{\tilde{E}_x\}_x$ is a noisy implementation of the POVM $\{E_x\}_x$. For the specific case of the all-zero computational basis state $|0\rangle\langle 0|^{\otimes n}$ we write $|0_n\rangle\rangle$ (with noisy version $|\tilde{0}_n\rangle\rangle$) and for the computational basis POVM $|x\rangle\langle x|$ we write $\langle\langle x|$ (with noisy version $\langle\langle \tilde{x}|$).

SUPPLEMENTARY NOTE 2. – TECHNICAL PRELIMINARIES ON REPRESENTATION THEORY

In this section we recall some basic facts of representation theory (of finite groups), and discuss generally how it applies to our work, with a particular focus on the representation theory of the Clifford group. For a more in depth introduction to representation theory, we recommend the standard textbook ref. [1].

Let $\mathbb{G}$ be a finite group and consider the space $\mathcal{M}_d$ of linear transformations of $\mathbb{C}^d$. A representation ω is a map $\omega : \mathbb{G} \to \mathcal{M}_d$ that preserves the group multiplication, i.e.,

$$\omega(g)\omega(h) = \omega(gh), \qquad \forall g, h \in \mathbb{G}. \tag{1}$$

We will require the operators $\omega(g)$ to be unitary as well (for finite groups this can always be done).

Reducible and irreducible representations. If there is a non-trivial subspace W of $\mathbb{C}^d$ such that for all vectors $w \in W$ we have

$$\omega(g)w \in W, \qquad \forall g \in \mathbb{G}, \tag{2}$$

then the representation ω is called *reducible*. The restriction of ω to the subspace W is also a representation, which we call a *sub-representation* of ω. If there are no non-trivial subspaces W such that eq. (2) holds the representation ω is called *irreducible*. We will generally reserve the letter σ to denote irreducible representations. Two representations ω, ω' of a group $\mathbb{G}$ are called *equivalent* if there exists an invertible linear map T such that

$$T\omega(g) = \omega'(g)T, \qquad \forall g \in \mathbb{G}. \tag{3}$$

We will denote this by $\omega \simeq \omega'$.

Sums, products, and Maschke's Lemma. We will make use of sums and products of representations. Given representations ω, ω', the maps

$$\omega \oplus \omega' : \mathbb{G} \to \mathcal{M}_d \oplus \mathcal{M}_{d'} : g \mapsto \omega(g) \oplus \omega'(g), \tag{4}$$

$$\omega \otimes \omega' : \mathbb{G} \to \mathcal{M}_d \otimes \mathcal{M}_{d'} : g \mapsto \omega(g) \otimes \omega'(g), \tag{5}$$

are again representations. They are, however, generally not irreducible (even if ω and ω' are). However, Maschke's Lemma ensures that every representation ω of a group can be uniquely written as a direct sum of irreducible representations, that is

$$\omega(g) \simeq \bigoplus_{\lambda \in S} \sigma_\lambda(g)^{\oplus n_\lambda}, \qquad \forall g \in \mathbb{G}, \tag{6}$$

where the index set S labels a subset of the irreducible representations of $\mathbb{G}$ and n_λ is an integer denoting the number of copies (or multiplicity) of σ_λ present in ω.

Averages of representations. Here we recall some standard results about averages over representations of finite groups. We will present these without proof, referring again to ref. [1] for a more detailed explanation. First is the basic statement that the average over any representation of a finite group is a projector (precisely onto the subspace on which the representation acts trivially):

Lemma 1. *Let ω be a representation of a group $\mathbb{G}$ then*

$$\mathop{\mathbb{E}}_{g\in\mathbb{G}} \omega(g) = P_{\text{inv}} \tag{7}$$

where P_{inv} is the projector onto the subspace left invariant under the action of $\omega(g)$, i.e., all vectors v s.t. $\omega(g)v = v$ for all $g \in \mathbb{G}$.

Second is a useful statement about the invariant subspaces of two-fold tensor powers of representations.

Lemma 2. *Let σ, σ' be real, irreducible, inequivalent, and non-trivial representations of a finite group $\mathbb{G}$. Then the representation $\sigma \otimes \sigma'$ has no invariant subspace, while the representation $\sigma^{\otimes 2}$ leaves the vector $v(P_\sigma)$ invariant, where $v(P_\sigma)$ is the vectorized projector onto the image of σ.*

Representation theory of the Clifford group. Here we give some basic facts about the representation theory of the Clifford group, which are used in the main text to derive the decay models for the multi-qubit and local Clifford UIRS protocols.

Lemma 3. *The Liouville representation of the n-qubit Clifford group $\mathbb{C}_n$ decomposes into two irreducible representations, in particular we have for all $g \in \mathbb{C}_n$:*

$$\omega(g) = \omega_{\text{triv}}(g) \oplus \omega_{\text{ad}}(g) \tag{8}$$

where $\omega_{\text{triv}}(g)$ has support on $\mathrm{Span}\{\tau_0\}$ and $\omega_{\text{ad}}(g)$ has support on the space of traceless matrices spanned by all normalized traceless Hermitian Pauli operators $\tau \in \mathbb{P}_0^*$.

This is a direct consequence of the 2-design property of the Clifford group. An early proof can be found in ref. [2]. A direct consequence is the following.

Lemma 4. *Let ω be the Liouville representation of the n-qubit Clifford group. Then we have*

$$\mathop{\mathbb{E}}_{g\in\mathbb{C}_n} \omega(g)^{\otimes 2} = |\tau_0^{\otimes 2}\rangle\!\rangle\langle\!\langle\tau_0^{\otimes 2}| + \frac{1}{2^{2n}-1} \sum_{\tau,\tau'\in\mathbb{P}_0^*} |\tau^{\otimes 2}\rangle\!\rangle\langle\!\langle{\tau'}^{\otimes 2}|. \tag{9}$$

We have similar statements for the local Clifford group.

Lemma 5. *The Liouville representation of the local Clifford group $\mathbb{C}_1^{\times n}$ on n qubits decomposes into 2^n mutually inequivalent irreducible representations*

$$\omega(g) = \bigoplus_{w\in\{0,1\}^n} \sigma_w(g) \tag{10}$$

where $\sigma_w(g)$ has support on $\mathrm{Span}\{\mathbb{P}_w^*\}$ with

$$\mathbb{P}_w = \left\{ \bigotimes_{i=1}^n \tau_i \;\;\|\;\; \tau_i = \tau_0 \text{ if } w_i = 0 \text{ and } \tau_i \in \{\tau_X, \tau_Y, \tau_Z\} \text{ if } w_i = 1 \right\}. \tag{11}$$

This is a direct result of the previous lemmas, applied to each of the n qubits individually. We also have the following statement.

Lemma 6. *Let ω be the Liouville representation of the local Clifford group on n qubits. Then we have*

$$\mathop{\mathbb{E}}_{g\in\mathbb{C}_1^{\times n}} \omega(g)^{\otimes 2} = \sum_{w\in\{0,1\}^n} \frac{1}{3^{|w|}} \left(\sum_{\tau\in\mathbb{P}_w^*} |\tau^{\otimes 2}\rangle\!\rangle \right) \left(\sum_{\tau'\in\mathbb{P}_w^*} \langle\!\langle{\tau'}^{\otimes 2}| \right), \tag{12}$$

where again

$$\mathbb{P}_w^* = \left\{ \bigotimes_{i=1}^n \tau_i \;\;\|\;\; \tau_i = \tau_0 \text{ if } w_i = 0 \text{ and } \tau_i \in \{\tau_X, \tau_Y, \tau_Z\} \text{ if } w_i = 1 \right\}. \tag{13}$$

SUPPLEMENTARY NOTE 3. – SIMULTANEOUSLY ESTIMATING MANY OBSERVABLES

Key to the results in this work is the following general statistics observation, which also powers state shadow estimation [3]. Let p be a probability distribution over some (finite) set $\mathcal{X}$, and let $\mathcal{A}$ be a set of observables, i.e., functions $f : \mathcal{X} \to \mathbb{R}$. Suppose we wish to estimate the vector of means $[\mathbb{E}_{\mathcal{X}}(f_A)]_{A\in\mathcal{A}}$ to some overall error ϵ. A surprising fact from mathematical statistics is that this is possible by drawing only $O(\log(|\mathcal{A}|)\mathbb{V}_{\max}(f_A)/\epsilon^2)$ samples from p where $\mathbb{V}_{\max}(f_A) = \max_{A\in\mathcal{A}} \mathbb{V}_X(f_A)$ is the maximal variance of the functions in $\mathcal{A}$. Doing this (without making strong assumptions on the observables f_A) requires the construction of so-called sub-Gaussian estimators (see refs. [4–6] for reviews) for the means $\mathbb{E}_{\mathcal{X}}(f_A)$. An example of such an estimator that is straightforward to compute is the *median-of-means estimator*, which has been used in state shadow estimation by ref. [3]. Following their notation, it involves gathering $S = NK$ samples $\{x_i\}_{i=1}^{NK}$ from the distribution p, where N, K are integers. For an observable f_A, one can then construct the estimator

$$\hat{f}_A = \text{Median}\left\{\frac{1}{N}\sum_{i=I}^{I+N-1} f_A(x_i) \parallel I \in \{1, N+1, 2N+1, \dots, (K-1)N+1\}\right\} \tag{14}$$

for the average $\mathbb{E}(f_A)$, splitting the data into K equally sized parts of size N. It can be shown that if we set

$$K = \lceil 2\log(2|\mathcal{A}|/\delta)\rceil, \tag{15}$$

$$N = \left\lceil \frac{34}{\epsilon^2}\mathbb{V}_{\max}(f_A)\right\rceil, \tag{16}$$

then we have

$$\max_{A\in\mathcal{A}} |\hat{f}_A - \mathbb{E}(f_A)| \le \epsilon, \tag{17}$$

with probability $1-\delta$. We can substitute ϵ to obtain the direct relation

$$\max_{A\in\mathcal{A}} |\hat{f}_A - \mathbb{E}(f_A)| \le \sqrt{\frac{68\mathbb{V}_{\max}(f_A)\log(2|\mathcal{A}|/\delta)}{NK}}, \tag{18}$$

in terms of the total number of samples NK. Hence, providing bounds on the maximal variance of a set of observables provides a rigorous guarantee on their estimation at any degree of confidence. Note however that the construction of the estimator is dependent on the level of confidence δ (through the setting of K). This is unfortunate, but it turns out to be impossible [6] to drop this requirement for sub-Gaussian estimators.

SUPPLEMENTARY NOTE 4. – GUARANTEES FOR THE UIRS PROTOCOL

In this section, we give the derivations of the general performance guarantees for the UIRS protocol summarized in the main text.

A. Fitting model

As we have argued in the main text, a useful class of sequence correlation functions is given by

$$f_A(x, \boldsymbol{g}) = \alpha\langle\langle E_x|\sigma(g_m)\prod_{i=1}^{m-1} A\sigma(g_i))|\rho\rangle\rangle\,, \tag{19}$$

where A is some fixed *probe super-operator*, α is a suitable normalization and $\sigma(g), \phi(g)$ are representations of the gate-set group $\mathbb{G}$. We begin by deriving the main result (eq. (5) in the main text) on the mean $k_A(m)$ in the UIRS protocol.

Theorem 7. *Let $k_A(m)$ be the outcome of an UIRS experiment with a correlation function as in eq. (19), over a gate-set $\mathbb{G}$. Then we have, under the assumption of gate-independent noise,*

$$k_A(m) = \text{Tr}\left(\Theta(\{E_x\}_x, \rho)[\Phi(A, \Lambda)]^{m-1}\right), \tag{20}$$

where Θ, Φ are matrices induced by the representation structure of $\omega(g)$. $\Phi(A, \Lambda)$ depends only on the between-gates noise channel $\Lambda := \Lambda_R\Lambda_L$ and the probe super-operator A, while Θ captures state preparation and measurement (SPAM) dependence. In particular, if ω contains n_σ copies of the representation σ then we have

$$\Phi_{i,j} = \frac{1}{|P_j|}\operatorname{Tr}(P_i A P_j \Lambda), \tag{21}$$

where P_i is the projector onto the ith copy of the representation σ inside ω.

The formulation of the result given in the main text directly follows from theorem 7 by additionally realizing that $f_A(x, \mathbf{g})$ regarded as a random variable pushing forward $p(x, \mathbf{g})$ is bounded and, thus, the corresponding mean estimator $\hat{k}_{f_A}(m)$ is unbiased and consistent. Correspondingly the median-of-mean estimator converges to the expected value of the mean.

Proof. Recall that $\phi(g) = \Lambda_L\omega(g)\Lambda_R$. Hence

$$\begin{aligned} k_A(m) &= \mathop{\mathbb{E}}_{\boldsymbol{g}\in\mathbb{G}^{\times m}} \sum_{x\in\{0,1\}^n} \alpha \langle\langle E_x|\sigma(g_m)\prod_{i=1}^{m-1} A\sigma(g_i))|\rho\rangle\rangle\langle\langle \tilde{E}_x|\prod_{i=1}^{m}\Lambda_L\omega(g_i)\Lambda_R|\tilde{\rho}\rangle\rangle \\ &= \mathop{\mathbb{E}}_{\boldsymbol{g}\in\mathbb{G}^{\times m}} \sum_{x\in\{0,1\}^n} \alpha\operatorname{Tr}\left(\left(|\rho\otimes\Lambda_R(\tilde{\rho})\rangle\rangle\langle\langle E_x\otimes\Lambda_L^*(\tilde{E}_x)|\right)\sigma(g_m)\otimes\omega(g_m)\prod_{i=1}^{m-1}\left((A\otimes\Lambda)(\sigma(g_i)\otimes\omega(g_i))\right)\right) \\ &= \sum_{x\in\{0,1\}^n}\alpha\operatorname{Tr}\left[\left(|\rho\otimes\Lambda_R(\tilde{\rho})\rangle\rangle\langle\langle E_x\otimes\Lambda_L^*(\tilde{E}_x)|\right)\left(\mathop{\mathbb{E}}_{g\in\mathbb{G}}(\sigma(g)\otimes\omega(g))(A\otimes\Lambda)\mathop{\mathbb{E}}_{g\in\mathbb{G}}(\sigma(g)\otimes\omega(g))\right)^{m-1}\right)\right], \end{aligned} \tag{22}$$

where we have used that the representation average is a projector (and thus equal to its square). Now note that we can write $\omega(g) = \sigma^{n_\sigma}(g)\oplus\omega'(g)$ where ω' is a representation that contains no copies of σ. From this and lemmas 1 and 2 given above we can see that

$$k_A(m) = \sum_{x\in\{0,1\}^n}\alpha\operatorname{Tr}\left(\left(|\rho\otimes\Lambda_R(\tilde{\rho})\rangle\rangle\langle\langle E_x\otimes\Lambda_L^*(\tilde{E}_x)|\right)\left(\sum_{i,i'=1}^{n_\sigma}\frac{1}{d_\sigma}|v(P_i)\rangle\rangle\langle\langle v(P_{i'})|(A\otimes\Lambda)\sum_{j,j'=1}^{n_\sigma}|v(P_j)\rangle\rangle\langle\langle v(P_{j'})|\right)^{m-1}\right), \tag{23}$$

where P_i is the projector on the i'th copy of σ in ω. Using the fact that

$$\langle\langle v(P)|A\otimes B|v(P')\rangle\rangle = \operatorname{Tr}(A^T P B P'), \tag{24}$$

and defining the matrices Θ, Φ appropriately, we obtain the theorem statement. ☐

B. Variance bound with the dynamic shadow norm

In order to bound the sampling complexity of the estimation of UIRS means $k_A(m)$ it is sufficient, through the use of median-of-means estimators, to obtain a bound on the variance of associated probability distribution. In the main text we did this by introducing the dynamic shadow norm. The dynamic shadow norm is formally defined as

$$\|A\|_{\mathrm{dyn},m} = \alpha^2\max_{\Lambda_R,\Lambda_L}\left|\sum_{x\in\mathcal{X}}\langle\langle E_x^{\otimes 2}\otimes\Lambda_L^*(\tilde{E}_x)|\left(P_\sigma^{(2)}(A^{\otimes 2}\otimes I)P_\sigma^{(2)}\right)^{m-1}|\rho^{\otimes 2}\otimes\Lambda_R(\tilde{\rho})\rangle\rangle\right|, \tag{25}$$

with

$$P_\sigma^{(2)} = \mathop{\mathbb{E}}_{g\in\mathbb{G}}\sigma(g)^{\otimes 2}\otimes\omega(g). \tag{26}$$

We prove the associated theorem:

Theorem 8 (Restatement of Theorem 1 in the main text)**.** *Consider an UIRS protocol (at sequence length m) with gate-set $\mathbb{G}$. Also consider a correlation function f_A with probe super-operator A. The (single-shot) variance of the associated mean estimator $\hat{k}_{f_A}(m)$ is bounded as*

$$\mathbb{V}_A(m) \le \|A\|_{\mathrm{dyn},m}\,. \tag{27}$$

Proof. The variance of a discrete random variable X assuming values $x \in \mathcal{X}$ with probability $p(x)$ is given by

$$\mathbb{V}(X) = \sum_{x\in\mathcal{X}} (x-\mu)^2 p(x), \tag{28}$$

with μ the expected value of X. We can therefore obtain an upper bound on the variance by simply considering

$$\mathbb{V}(X) \leq \sum_{x\in\mathcal{X}} (x)^2 p(x). \tag{29}$$

Thus, we have that

$$\mathbb{V}_A(m) = \mathbb{V}(X_A(m)) \leq \alpha^2 \mathop{\mathbb{E}}_{\boldsymbol{g}\in\mathbb{G}^{\times m}} \sum_{x\in\mathcal{X}} \langle\langle E_x|\sigma(g_m)\prod_{i=1}^{m-1} A\sigma(g_i))|\rho\rangle\rangle^2 \langle\langle \tilde{E}_x|\prod_{i=1}^{m}\phi(g_i)|\tilde{\rho}\rangle\rangle. \tag{30}$$

Using the identities $\mathrm{Tr}(A\otimes B) = \mathrm{Tr}(A)\,\mathrm{Tr}(B)$ and $AB\otimes AB = A^{\otimes 2}B^{\otimes 2}$ we obtain

$$\mathbb{V}_A(m) \leq \alpha^2 \sum_{x\in\mathcal{X}} \mathrm{Tr}\left((|\rho^{\otimes 2}\otimes\Lambda_R(\tilde{\rho})\rangle\rangle\langle\langle E_x^{\otimes 2}\otimes\Lambda_L^*(\tilde{E}_x)|) \mathop{\mathbb{E}}_{g\in\mathbb{G}} \sigma(g)^{\otimes 2}\otimes\omega(g) \Big(\mathop{\mathbb{E}}_{g\in\mathbb{G}} (A\sigma(g))^{\otimes 2}\otimes(\Lambda\omega(g)) \Big)^{m-1} \right). \tag{31}$$

Maximizing over Λ_R, Λ_L and recalling the definition

$$\|A\|_{\mathrm{dyn},m} := \alpha^2 \max_{\Lambda_R,\Lambda_L} \left| \sum_{x\in\mathcal{X}} \langle\langle E_x^{\otimes 2}\otimes\Lambda_L^*(\tilde{E}_x)| \big(P_\sigma^{(2)}(A^{\otimes 2}\otimes\Lambda)P_\sigma^{(2)}\big)^{m-1} |\rho^{\otimes 2}\otimes\Lambda_R(\tilde{\rho})\rangle\rangle \right|, \tag{32}$$

of the shadow norm completes the argument.

□

SUPPLEMENTARY NOTE 5. – SHADOW NORM BOUND FOR LOCAL CLIFFORD UIRS

In this section, we consider the UIRS protocol with the local Clifford group $\mathbb{C}_1^{\times n}$. We will model the noisy implementation of any given Clifford by $\Lambda_L\omega(g)\Lambda_R$ and we will denote $\Lambda_R\Lambda_L =: \Lambda$ for brevity. In the main text we stated the following theorem:

Theorem 9 (Restatement of Theorem 4 in the main text)**.** *Consider the local Clifford UIRS protocol. Let $\phi(g) = \Lambda_L\omega(g)\Lambda_R$ be a noisy implementation of the local Clifford group on n qubits and $A = P_wAP_w$ be a probe super-operator with $|w| = k$ for k a fixed integer. Also let $\tilde{0}_n$ be a noisy implementation of the all-zero state and $\{\tilde{x}\}_x$ the noisy computational basis POVM). The shadow norm of the random variable $X_A(m)$ is upper bounded independently of the number of qubits n and sequence length m. In particular, it holds that*

$$\|A\|_{\mathrm{dyn},m} \leq 2^k 3^{2k} \big(3^{-k}\,\mathrm{Tr}(AA^\dagger)\big)^{m-1}. \tag{33}$$

Proof. We begin from the general expression of the shadow norm, which can be written as

$$\begin{aligned}
\|A\|_{\mathrm{dyn},m} &= \max_{\Lambda_R,\Lambda_L} 3^{2k}2^{2n} \sum_{x\in\{0,1\}^n} \langle\langle x^{\otimes 2}\otimes\tilde{x}|(\mathbb{I}^{\otimes 2}\otimes\Lambda_L)\left[\mathop{\mathbb{E}}_{g_{[1,n]}} \omega(g_{[1,n]})^{\otimes 3}(A^{\otimes 2}\otimes\Lambda) \mathop{\mathbb{E}}_{g'_{[1,n]}} \omega(g'_{[1,n]})^{\otimes 3}\right]^m (\mathbb{I}^{\otimes 2}\otimes\Lambda_R)|0_n^{\otimes 2}\otimes\tilde{0}_n\rangle\rangle \\
&= \max_{\Lambda_R,\Lambda_L} 3^{2k}2^{2n} \mathop{\mathbb{E}}_{g^{(1)}_{[1,n]},\dots,g^{(m)}_{[1,n]}} \sum_{x\in\{0,1\}^n} \langle\langle x^{\otimes 2}|\omega(g^{(m)}_{[1,n]})^{\otimes 2}\prod_{i=1}^{m-1}\big(A^{\otimes 2}\omega(g^{(i)}_{[1,n]})^{\otimes 2}\big)|0_n^{\otimes 2}\rangle\rangle \\
&\qquad\qquad \times \langle\langle\tilde{x}|\Lambda_L\omega(g^{(m)}_{[1,n]})\prod_{i=1}^{m-1}\big(\Lambda\omega(g^{(i)}_{[1,n]})\big)\Lambda_R|\tilde{0}_n\rangle\rangle,
\end{aligned}$$

where $\{\langle\langle\tilde{x}|\}_x$ and $|\tilde{0}_n\rangle\rangle$ are the noisy measurement POVM and the noisy initial state, respectively.

We now make use of the fact that A is assumed to be supported on only a single irreducible representation denoted by $k\in\{0,1\}^n$ i.e., $A = P_kAP_k$ where P_k is the projector onto that irreducible representation. Without loss of generality we will

here set k to be the all 1 bit string on the first k bits and 0 on the remaining $n-k$ bits. The projector P_w acts as $|\tau_0\rangle\rangle\langle\langle\tau_0|$ on these last $n-k$ qubits. Since

$$\omega(g)|\tau_0\rangle\rangle = |\tau_0\rangle\rangle, \tag{34}$$

we see that in $\langle\langle x^{\otimes 2}|\omega(g^{(m)}_{[1,n]})^{\otimes 2}\prod_{i=1}^{m-1}\left(A^{\otimes 2}\omega(g^{(i)}_{[1,n]})^{\otimes 2}\right)|0_n^{\otimes 2}\rangle\rangle$ we can absorb the action of the local Clifford group on the last $n-k$ qubits. Hence, the local Cliffords $g^{(i)}_{[k+1,n]}$ only act by a single conjugation, i.e.,

$$\begin{aligned}\|A\|_{\mathrm{dyn},m} = \max_{\Lambda_R,\Lambda_L} 3^{2k}2^{2n} \mathop{\mathbb{E}}_{g^{(1)}_{[1,n]},\ldots,g^{(m)}_{[1,n]}} \sum_{x\in\{0,1\}^n} &\langle\langle x^{\otimes 2}|\omega(g^{(m)}_{[1,k]})^{\otimes 2}\prod_{i=1}^{m-1}\left(A^{\otimes 2})\omega(g^{(i)}_{[1,k]})^{\otimes 2}\right)|0_n^{\otimes 2}\rangle\rangle \\ &\times \langle\langle \Lambda_L^*(\tilde{x})|\omega(g^{(m)}_{[1,k]})\otimes\omega(g^{(m)}_{[k+1,n]})\prod_{i=1}^{m-1}\left(\Lambda\omega(g^{(i)}_{[1,k]})\omega(g^{(i)}_{[k+1,n]})\right)|\Lambda_R(\tilde{0}_n)\rangle\rangle \\ &\times \left[\langle\langle x_{[k+1,n]}|\tau_{0\,[k+1,n]}\rangle\rangle\langle\langle\tau_{0\,[k+1,n]}|0_{n\,[k+1,n]}\rangle\rangle\right]^2.\end{aligned} \tag{35}$$

Now we use the fact that

$$\langle\langle x_{[k+1,n]}|\tau_{0\,[k+1,n]}\rangle\rangle = 2^{(k-n)/2}, \qquad x_{[k+1,n]}\in\{0,1\}^{n-k}, \tag{36}$$

and

$$\mathop{\mathbb{E}}_{g_{[k+1,n]}} \omega(g_{[k+1,n]}) = |\tau_{0\,[k+1,n]}\rangle\rangle\langle\langle\tau_{0\,[k+1,n]}|, \tag{37}$$

we end up with

$$\begin{aligned}\|A\|_{\mathrm{dyn},m} = \max_{\Lambda_R,\Lambda_L} 3^{2k}2^{2k} \mathop{\mathbb{E}}_{g^{(1)}_{[1,k]},\ldots,g^{(m)}_{[1,k]}} \sum_{x_{[1,k]}\in\{0,1\}^k} &\langle\langle x^{\otimes 2}_{[1,k]}|\omega(g^{(m)}_{[1,k]})^{\otimes 2}\prod_{i=1}^{m-1}\left(A^{\otimes 2}\omega(g^{(i)}_{[1,k]})^{\otimes 2}\right)|0_k^{\otimes 2}\rangle\rangle \\ \times \sum_{x_{[k+1,n]}\in\{0,1\}^{n-k}} &2^{k-n}\langle\langle \mathrm{Tr}_{k+1,n}(\Lambda_L^*(\tilde{x}))|\omega(g^{(m)}_{[1,k]})\prod_{i=1}^{m-1}\left(\Lambda\omega(g^{(i)}_{[1,k]})\right)|\mathrm{Tr}_{k+1,n}(\Lambda_R(\tilde{0}_n))\rangle\rangle.\end{aligned} \tag{38}$$

Obviously $|\tilde{\rho}_k\rangle\rangle := |\mathrm{Tr}_{k+1,n}(\Lambda_R(\tilde{0}_n))\rangle\rangle$ is a k-qubit state and, moreover, we have

$$\begin{aligned}\sum_{x_{[1,k]}\in\{0,1\}^k}\tilde{E}_{x_{[1,k]}} &:= \sum_{x_{[1,k]}\in\{0,1\}^k}\left(2^{k-n}\sum_{x_{[k+1,n]}\in\{0,1\}^{n-k}}\mathrm{Tr}_{k+1,n}(\Lambda_L^*(\tilde{x}))\right) \\ &= 2^{k-n}\sum_{x\in\{0,1\}^n}\mathrm{Tr}_{k+1,n}(\Lambda_L^*(\tilde{x})) \\ &= 2^{k-n}\,\mathrm{Tr}_{k+1,n}(\Lambda_L^*(\mathbb{I})) \\ &= 2^{k-n}\,\mathrm{Tr}_{k+1,n}(\mathbb{I}) = \mathbb{I}_{[1,k]},\end{aligned} \tag{39}$$

which makes $\{\tilde{E}_{x_{[1,k]}}\}_{x_{[1,k]}}$ a k-qubit POVM. Hence, we have

$$\begin{aligned}\|A\|_{\mathrm{dyn},m} = \max_{\Lambda_R,\Lambda_L} 3^{2k}2^{2k} \mathop{\mathbb{E}}_{g^{(1)}_{[1,k]},\ldots,g^{(m)}_{[1,k]}} \sum_{x_{[1,k]}\in\{0,1\}^k} &\langle\langle x^{\otimes 2}_{[1,k]}|\omega(g^{(m)}_{[1,k]})^{\otimes 2}\prod_{i=1}^{m-1}\left(A^{\otimes 2}\omega(g^{(i)}_{[1,k]})^{\otimes 2}\right)|0_k^{\otimes 2}\rangle\rangle \\ &\times \langle\langle \tilde{E}_{x_{[1,k]}}|\omega(g^{(m)}_{[1,k]})\prod_{i=1}^{m-1}\left(\Lambda_k\omega(g^{(i)}_{[1,k]})\right)|\tilde{\rho}_k\rangle\rangle,\end{aligned} \tag{40}$$

where Λ_k is the k-qubit marginal of Λ. At this point, we see that the shadow norm is bounded independently of the number of qubits n and only depends on the dimension of the irreducible representation which we assume A to have support on (it is a

function of k only). As we can see, we are left with a third moment calculation over $\mathbb{C}_1^{\times k}$. However, for the sake of obtaining an upper bound we simply note that

$$\langle\!\langle \tilde{E}_{x_{[1,k]}}|\omega(g^{(m)}_{[1,k]})\prod_{i=1}^{m-1}\big(\Lambda_k\omega(g^{(i)}_{[1,k]})\big)|\tilde{\rho}_k\rangle\!\rangle \in [0,1], \tag{41}$$

since Λ_k is a quantum channel. We, therefore, can (using the invariance of the Haar measure) simplify the bound to

$$\|A\|_{\mathrm{dyn},m} \le 3^{2k}2^{3k}\mathop{\mathbb{E}}_{g_{[1,k]}}\mathop{\mathbb{E}}_{g'_{[1,k]}}\langle\!\langle 0^{\otimes 2}_{[1,k]}|\left[\omega(g_{[1,k]})^{\otimes 2}A^{\otimes 2}\omega(g'_{[1,k]})^{\otimes 2}\right]^{m-1}|0^{\otimes 2}_{[1,k]}\rangle\!\rangle. \tag{42}$$

Again using the fact that A is taken to only have overlap with a single irreducible representation, lemma 6 and the fact that $\langle\!\langle 0|\tau_i\rangle\!\rangle = 1/\sqrt{2}$ if and only if $\tau_i = \tau_0$ or τ_Z (and zero otherwise) we have

$$\begin{aligned}\|A\|_{\mathrm{dyn},m} &\le 3^{2k}2^{3k}\left[\frac{\mathrm{Tr}(AA^\dagger)}{3^k}\right]^{m-1}\langle\!\langle 0^{\otimes 2k}|\tau_Z^{\otimes 2k}\rangle\!\rangle^2 \\ &= 3^{2k}2^{k}\left[\frac{\mathrm{Tr}(AA^\dagger)}{3^k}\right]^{m-1},\end{aligned} \tag{43}$$

which is what we set out to prove. □

SUPPLEMENTARY NOTE 6. – SHADOW NORM BOUND FOR MULTI-QUBIT CLIFFORD UIRS

In this section, we will go into the details of the shadow norm bound calculations for the multi-qubit Clifford group UIRS protocol. Concretely, we prove the following theorem.

Theorem 10 (Restatement of Theorem 3 in the main text)**.** *Consider the n-qubit Clifford UIRS protocol and let $A = P_{\mathrm{ad}}AP_{\mathrm{ad}}$ be a probe super-operator restricted to the traceless subspace. Also let $\tilde{0}_n$ be a noisy implementation of the all-zero state and $\{\tilde{x}\}_{x\in\{0,1\}^n}$ the noisy computational basis POVM). The associated shadow norm is upper bounded as*

$$\|A\|_{\mathrm{dyn},m} \le 11\,u(A)\Big(r(A)^{m-2} + \left[2(m-2)^2 r(A)^{m-3}\right]\max\left\{11u(A),(11\,u(A))^2\right\}\Big), \tag{44}$$

with

$$r(A) = u(A)(1 + 16\,2^{-n/3}), \tag{45}$$

and where $u(A) = \mathrm{Tr}(AA^\dagger)/(2^{2n}-1)$ is the unitarity of A.

Restricting to $m > 0$ and using that $r(A) \ge u(A)$ yields the simplified statement in the main text.

Proof. Recall that the dynamic shadow norm for the multi-qubit Clifford group is given by

$$\|A\|_{\mathrm{dyn},m} = \max_{\Lambda_R,\Lambda_L}(2^n+1)^2\sum_{x\in\{0,1\}^n}\mathrm{Tr}\left[|0_n^{\otimes 2}\otimes\Lambda_R(\tilde{0}_n)\rangle\!\rangle\langle\!\langle x^{\otimes 2}\otimes\Lambda_L^*(\tilde{x})|\mathop{\mathbb{E}}_{g\in\mathbb{G}}\sigma_{\mathrm{ad}}(g)^{\otimes 2}\otimes\omega(g)\left[\mathop{\mathbb{E}}_{g\in\mathbb{G}}(A\sigma_{\mathrm{ad}}(g))^{\otimes 2}\otimes(\Lambda\omega(g))\right]^{m-1}\right], \tag{46}$$

where $\Lambda := \Lambda_R\Lambda_L$ is the noise in-between subsequent gates. We can provide a concrete resolution for the third moment by noting that $\mathbb{C}_n$ is a 3-design [7], and, hence, its third moment follows that of the unitary group $U(2^n)$, which is fully determined (for $n \ge 2$) by Schur-Weyl duality. In particular, we have

$$\mathop{\mathbb{E}}_{c\in\mathbb{C}_n}\sigma_{\mathrm{ad}}(c)^{\otimes 2}\otimes\omega(c) = P_{\mathrm{ad}}^{\otimes 2}\otimes\mathbb{I}\left[\mathop{\mathbb{E}}_{c\in\mathbb{C}_n}\omega(c)^{\otimes 3}\right]P_{\mathrm{ad}}^{\otimes 2}\otimes\mathbb{I} = \sum_{\pi,\pi'\in S_3}W_{\pi,\pi'}(P_{\mathrm{ad}}^{\otimes 2}\otimes\mathbb{I})|\pi\rangle\!\rangle\langle\!\langle\pi'|(P_{\mathrm{ad}}^{\otimes 2}\otimes\mathbb{I}), \tag{47}$$

where the matrices π permute copies of the base Hilbert space, i.e.,

$$\pi\,|i_1,i_2,i_3\rangle = |i_{\pi(1)},i_{\pi(2)},i_{\pi(3)}\rangle\,. \tag{48}$$

The Weingarten matrix

$$W = \frac{1}{2^n(2^{2n}-1)(2^{2n}-4)} \begin{pmatrix} 2^{2n}-2 & -2^n & -2^n & -2^n & 2 & 2 \\ -2^n & 2^{2n}-2 & 2 & 2 & -2^n & -2^n \\ -2^n & 2 & 2^{2n}-2 & 2 & -2^n & -2^n \\ -2^n & 2 & 2 & 2^{2n}-2 & -2^n & -2^n \\ 2 & -2^n & -2^n & -2^n & 2^{2n}-2 & 2 \\ 2 & -2^n & -2^n & -2^n & 2 & 2^{2n}-2 \end{pmatrix} \tag{49}$$

can be explicitly written down in the basis $\{e, (12), (23), (13), (123), (132)\}$. Now defining the matrices

$$\Omega_{\pi',\pi} = \langle\!\langle \pi' | A^{\otimes 2} \otimes \Lambda | \pi \rangle\!\rangle, \tag{50}$$

and

$$[\Theta_x]_{\pi,\pi'} = (2^n+1)^2 \langle\!\langle \pi | 0_n^{\otimes 2} \otimes \Lambda_R(\tilde{0}_n) \rangle\!\rangle \langle\!\langle x^{\otimes 2} \otimes \Lambda_L^*(\tilde{x}) | \pi' \rangle\!\rangle, \tag{51}$$

we get

$$\|A\|_{\mathrm{dyn},m} = \max_{\Lambda_R,\Lambda_L} \sum_{x\in\{0,1\}^n} \mathrm{Tr}(\Theta_x (W\Omega)^{m-1} W). \tag{52}$$

We begin by analyzing the matrix Ω. Note that $A(\mathbb{I}) = A^\dagger(\mathbb{I}) = 0$, since A is supported only on the space of traceless matrices by construction. This means that $\Omega_{\pi,\pi'}$ is zero unless $\pi, \pi' \in \{(12), (123), (132)\}$. Thus we can write $\Omega = P^\dagger \hat{\Omega} P$ with P the restriction from $\mathrm{Span}\{e, (12), (23), (13), (123), (132)\}$ to $\mathrm{Span}\{(12), (123), (132)\}$ and

$$\hat{\Omega} = \begin{pmatrix} 2^n \,\mathrm{Tr}(AA^\dagger) & \mathrm{Tr}(AA^\dagger) & \mathrm{Tr}(AA^\dagger) \\ \mathrm{Tr}(J_u(A)^2 \mathbb{I} \otimes \Lambda(\mathbb{I})) & \mathrm{Tr}(J_u(A)^2 J_u(\Lambda)) & \mathrm{Tr}(J_u(AT)^2 J_u(\Lambda T)) \\ \mathrm{Tr}(J_u(A)^2 \mathbb{I} \otimes \Lambda(\mathbb{I})) & \mathrm{Tr}(J_u(TA)^2 J_u(T\Lambda)) & \mathrm{Tr}(J_u(A)^2 J_u(\Lambda)) \end{pmatrix},$$

where we have used $\Lambda^\dagger(\mathbb{I}) = \mathbb{I}$, J_u denotes the unnormalized Choi-isomorphism ($J_u(A) = \mathbb{I} \otimes A(|v(\mathbb{I})\rangle\langle v(\mathbb{I})|$ with v the column-stacking vectorization map) and T is the (non-CP) transposition map. This can be derived directly from the definition of Ω and some diagram chasing. Before we continue, we establish some facts about the entries of $\hat{\Omega}$. We begin by noting that for the off-diagonal term $\hat{\Omega}_{(123),(132)}$, we have

$$\mathrm{Tr}(J_u(AT)^2 J_u(\Lambda T)) = \mathrm{Tr}(J_u(TA)^2 J_u(T\Lambda)) = \mathrm{Tr}(J_u(A^\dagger T)^2 J_u(\Lambda^\dagger T)). \tag{53}$$

This follows from three facts: (1) $A^{\otimes 2}$ commutes with the super-operator $L_{(12)}$ defined as left-multiplication with the matrix (12), (2) $L_{(12)}((123)) = (132)$ and (3) the trace is invariant under Hermitian conjugation. One can also see this graphically through the following series of tensor manipulations: as

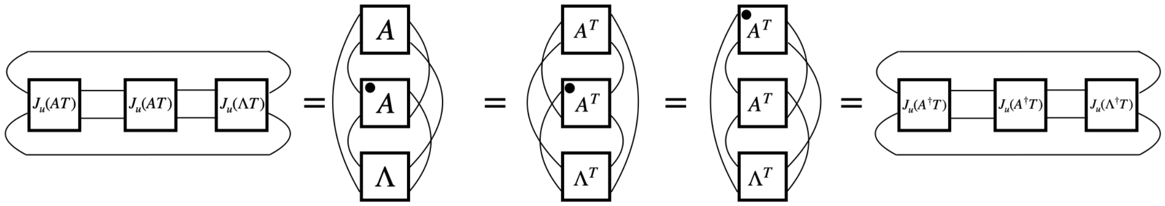

where we have used that the Liouville representation of a dual super-operator $\Lambda^\dagger$ is given by the transpose of the Liouville representation of Λ. For clarity we marked for clarity one of the copies of A with a dot. The rightmost side of eq. (53) is amenable to a bound of the form

$$\begin{aligned} |\,\mathrm{Tr}(J_u(A^\dagger T)^2 J(\Lambda^\dagger T))| &\le \mathrm{Tr}(J_u(A^\dagger T)^2) \left\|J_u(\Lambda^\dagger T)\right\|_\infty \\ &= \mathrm{Tr}(AA^\dagger) \left\|J_u(\Lambda^\dagger T)\right\|_\infty \\ &= \mathrm{Tr}(AA^\dagger) \max_{\|Q\|_1=1} |\,\mathrm{Tr}(Q\,\mathbb{I} \otimes \Lambda^\dagger(|v(\mathbb{I})\rangle\langle v(\mathbb{I})|^{T_B}))| \\ &= \mathrm{Tr}(AA^\dagger) \max_{\|Q\|_1=1} |\,\mathrm{Tr}(\mathbb{I} \otimes \Lambda(Q)(|v(\mathbb{I})\rangle\langle v(\mathbb{I})|^{T_B}))| \\ &\le \mathrm{Tr}(AA^\dagger) \left\|(|v(\mathbb{I})\rangle\langle v(\mathbb{I})|^{T_B})\right\|_\infty \\ &\le \mathrm{Tr}(AA^\dagger), \end{aligned} \tag{54}$$

where we have used Hölders inequality twice, the fact that A is Hermiticity preserving, and the fact that Λ is $1 \to 1$-norm contractive (by grace of being CPTP). Finally the Schatten ∞-norm of $(|v(\mathbb{I})\rangle \langle v(\mathbb{I})|^{T_B})$ is easily seen to be one. Furthermore, for the diagonal elements $\hat{\Omega}_{(123),(123)} = \hat{\Omega}_{(132),(132)}$, we have

$$\mathrm{Tr}(J_u(A)^2 J_u(\Lambda)) \leq \mathrm{Tr}(AA^\dagger) \left\| J_u(\Lambda) \right\|_\infty \leq 2^n \, \mathrm{Tr}(AA^\dagger) \tag{55}$$

again using Hölder's inequality and $\left\| J_u(\Lambda) \right\|_\infty \leq 2^n$ (note again that J_u is unnormalized). Finally, we consider the other off-diagonal term $\hat{\Omega}_{(123),(12)} = \hat{\Omega}_{(132),(12)}$. If Λ is unital (and thus $\Lambda(\mathbb{I}) = \mathbb{I}$), we see that $\hat{\Omega}_{(123),(12)} = \mathrm{Tr}(AA^\dagger)$, and in general we have

$$\mathrm{Tr}(J_u(A)^2 \mathbb{I} \otimes \Lambda(\mathbb{I})) \leq \mathrm{Tr}(AA^\dagger) \left\| \mathbb{I} \otimes \Lambda(\mathbb{I}) \right\|_\infty \leq 2^n \, \mathrm{Tr}(AA^\dagger). \tag{56}$$

Note that if Λ is unital the matrix $\hat{\Omega}$ is symmetric, however, for general channels this is not the case.

With these expressions we can move on to calculate the dynamic shadow norm

$$\|A\|_{\mathrm{dyn},m} = \max_{\Lambda_R, \Lambda_L} \sum_{x \in \{0,1\}^n} \mathrm{Tr}(\Theta_x (W\Omega)^{m-1} W) \leq \left\| \hat{\Omega} P W \Big(\sum_{x \in \{0,1\}^n} \Theta_x \Big) W P^\dagger \right\|_\infty \left\| \hat{\Omega} \hat{W}^{m-2} \right\|_\infty, \tag{57}$$

where $\hat{W} = PWP^\dagger$ and the norm is the standard operator (or Schatten $\infty-$) norm. To bound the norm-of-power factor (the 'dynamic' part of the shadow norm) in the above equation we need two useful lemmas about small non-normal matrices.

Lemma 11. *Let A, B be complex $d \times d$ matrices with spectral radius $s(A), s(B)$. We have that*

$$|s(A) - s(B)| \leq (\|A\|_\infty + \|B\|_\infty)^{1-1/d} \|A - B\|_\infty^{1/d}. \tag{58}$$

Lemma 12. *Let A be a complex $d \times d$ matrix with spectral radius $s(A)$. We have that*

$$\|A^m\|_\infty \leq s(A)^m + (d-1) m^{d-1} \max\left\{ s(A)^{m-d+1}, s(A)^{m-1} \right\} \max\left\{ 2\|A\|_\infty, (2\|A\|_\infty)^{d-1} \right\}. \tag{59}$$

Lemma 11 is a trivial consequence of ref. [8, Thm VIII.1.1], and we will prove lemma 12 in at the end of this section. Next, we define matrices

$$\hat{\Omega}^0 = \begin{pmatrix} 2^n \, \mathrm{Tr}(AA^\dagger) & 0 & 0 \\ \mathrm{Tr}(J_u(A)^2 \mathbb{I} \otimes \Lambda(\mathbb{I})) & \mathrm{Tr}(J_u(A)^2 J_u(\Lambda)) & \mathrm{Tr}(J_u(AT)^2 J_u(\Lambda T)) \\ \mathrm{Tr}(J_u(A)^2 \mathbb{I} \otimes \Lambda(\mathbb{I})) & \mathrm{Tr}(J_u(TA)^2 J_u(T\Lambda)) & \mathrm{Tr}(J_u(A)^2 J_u(\Lambda)) \end{pmatrix}, \qquad \hat{D} = \frac{2^{2n} - 2}{2^n (2^{2n} - 1)(2^{2n} - 4)} \mathbb{I}. \tag{60}$$

We have

$$\hat{\Omega}^0 \hat{D} = \hat{\Omega} \hat{W} + (\hat{\Omega}^0 - \hat{\Omega}) \hat{D} + \hat{\Omega} (\hat{D} - \hat{W}). \tag{61}$$

We can directly calculate the spectrum of $\hat{\Omega}^0 \hat{D}$ as being

$$\begin{aligned} \mathrm{Spec}(\hat{\Omega}^0 \hat{D}) = \Bigg\{ & \frac{2^n (2^{2n} - 2) \, \mathrm{Tr}(AA^\dagger)}{2^n (2^{2n} - 1)(2^{2n} - 4)}, \frac{(2^{2n} - 2)}{2^n (2^{2n} - 1)(2^{2n} - 4)} \big(\mathrm{Tr}(J_u(A)^2 J_u(\Lambda)) + \mathrm{Tr}(J_u(TA)^2 J_u(T\Lambda)) \big) \\ & , \frac{(2^{2n} - 2)}{2^n (2^{2n} - 1)(2^{2n} - 4)} \big(\mathrm{Tr}(J_u(A)^2 J_u(\Lambda)) - \mathrm{Tr}(J_u(TA)^2 J_u(T\Lambda)) \big) \Bigg\}. \end{aligned} \tag{62}$$

Using the definition of unitarity we see that the first eigenvalue is upper bounded by

$$\frac{2^n (2^{2n} - 2) \, \mathrm{Tr}(AA^\dagger)}{2^n (2^{2n} - 1)(2^{2n} - 4)} = \frac{2^n (2^{2n} - 2)(2^{2n} - 1)}{2^n (2^{2n} - 1)(2^{2n} - 4)} u(A) \leq (1 + 3\, 2^{-2n}) u(A), \tag{63}$$

for $n \geq 2$. Note that the constant 3 in the last inequality is somewhat loose. Furthermore, using eqs. (54) and (55) we can also provide bounds for the other two eigenvalues, to get

$$\begin{aligned} \frac{(2^{2n} - 2)}{2^n (2^{2n} - 1)(2^{2n} - 4)} \big(\mathrm{Tr}(J_u(A)^2 J_u(\Lambda)) \pm \mathrm{Tr}(J_u(TA)^2 J_u(T\Lambda)) \big) &\leq \frac{(2^{2n} - 2)(2^n + 1)}{2^n (2^{2n} - 1)(2^{2n} - 4)} \mathrm{Tr}(AA^\dagger) \\ &= \frac{(2^{2n} - 2)(2^n + 1)}{2^n (2^{2n} - 4)} u(A) \\ &\leq (1 + 4\, 2^{-n}) u(A), \end{aligned} \tag{64}$$

where again the constant 4 is an overestimate. Hence, the spectral radius of $\hat{\Omega}^0$ is bounded by $u(A)$ up to a small multiplicative correction, i.e.

$$s(\hat{\Omega}^0) \leq (1 + 4\,2^{-n})u(A). \tag{65}$$

The plan is now to leverage lemma 11 to bound the spectral radius of $\hat{\Omega}\hat{W}$. To do this we first need to bound the norm (from eq. (61))

$$\left\|\hat{\Omega}^0\hat{D} - \hat{\Omega}\hat{W}\right\|_\infty \leq \left\|(\hat{\Omega} - \hat{\Omega}^0)\right\|_\infty \left\|\hat{D}\right\|_\infty + \left\|\hat{\Omega}\right\|_\infty \left\|(\hat{D} - \hat{W})\right\|_\infty. \tag{66}$$

We can bound the terms on the RHS in a straightforward manner (using the definition eq. (49)),

$$\left\|(\hat{D} - \hat{W})\right\|_\infty \leq \left\|(\hat{D} - \hat{W})\right\|_{HS} = \frac{\sqrt{4\,2^{2n} + 8}}{2^n(2^{2n} - 1)(2^{2n} - 4)}, \tag{67}$$

and (using the definition of $\hat{\Omega}^0$)

$$\left\|(\hat{\Omega} - \hat{\Omega}^0)\right\|_\infty \leq (2^{2n} - 1)u(A), \tag{68}$$

and finally

$$\left\|\hat{\Omega}\right\|_\infty \leq \left\|\hat{\Omega}\right\|_{HS} \leq 3\,2^n(2^{2n} - 1)u(A), \tag{69}$$

$$\left\|\hat{D}\right\|_\infty = \frac{2^{2n} - 2}{2^n(2^{2n} - 1)(2^{2n} - 4)}. \tag{70}$$

Plugging this all back in we get

$$\left\|\hat{\Omega}^0\hat{D} - \hat{\Omega}\hat{W}\right\|_\infty \leq u(A)\frac{3\,2^n(2^{2n} - 1)\sqrt{4\,2^{2n} + 8} + (2^{2n} - 2)(2^{2n} - 1)}{2^n(2^{2n} - 1)(2^{2n} - 4)} \leq 3\,2^{-n}u(A). \tag{71}$$

Moreover, we have that

$$\left\|\hat{\Omega}^0\hat{D}\right\|_\infty \leq \frac{9\,u(A)2^n(2^{2n} - 1)(2^n - 2)}{2^n(2^{2n} - 1)(2^{2n} - 4)} \leq 11\,u(A), \tag{72}$$

for $n \geq 3$, and by the same argument

$$\left\|\hat{\Omega}\hat{W}\right\|_\infty \leq 11\,u(A). \tag{73}$$

Hence, through lemma 11, the spectral radius difference can be bounded as

$$|s(\hat{\Omega}^0\hat{D}) - s(\hat{\Omega}\hat{W})| \leq \big(22u(A)\big)^{2/3}\big(3\,2^{-n}u(A)\big)^{1/3} \leq 12\,2^{-n/3}u(A), \tag{74}$$

and the spectral radius of $\hat{\Omega}\hat{W}$ is thus bounded by (using the above equation and eq. (65))

$$s(\hat{\Omega}\hat{W}) \leq (1 + 4\,2^{-n} + 12\,2^{-n/3})u(A). \tag{75}$$

We can plug this into lemma 12 to obtain

$$\left\|\hat{\Omega}\hat{W}^{m-2}\right\| \leq (u(A)(1 + 16\,2^{-n/3}))^{m-2} + \left[2(m-2)^2(u(A)(1 + 16\,2^{-n/3}))^{m-3}\right] \max\left\{11u(A), (11\,u(A))^2\right\}, \tag{76}$$

which takes care of the ‘dynamic’ part of the dynamic shadow norm. To bound the SPAM contribution we can observe

$$\left\|\hat{\Omega}PW(\sum_{x\in\{0,1\}^n}\Theta_x)WP^\dagger\right\|_\infty \leq \left\|\hat{\Omega}\right\|_{HS}\|W\|_{HS}^2\left\|\sum_{x\in\{0,1\}^n}\Theta_x\right\|_{HS}. \tag{77}$$

Of these only $\|\sum_{x\in\{0,1\}^n}\Theta_x\|_{HS}$ has not been considered. From a straightforward calculation (using that $\Lambda_R(\tilde{0}_n)$ is a state and $\{\Lambda_L^*(\tilde{x})\}_x$ a POVM) one sees that $\sum_{x\in\{0,1\}^n}\Theta_x = 2^n(2^n+1)^2 v_1 v_2^T$ with

$$v_1 := \Big(1, 1, \mathop{\mathbb{E}}_{x\in\{0,1\}^n} \langle\!\langle x|\Lambda_L^*(\tilde{x})\rangle\!\rangle, \mathop{\mathbb{E}}_{x\in\{0,1\}^n} \langle\!\langle x|\Lambda_L^*(\tilde{x})\rangle\!\rangle, \mathop{\mathbb{E}}_{x\in\{0,1\}^n} \langle\!\langle x|\Lambda_L^*(\tilde{x})\rangle\!\rangle, \mathop{\mathbb{E}}_{x\in\{0,1\}^n} \langle\!\langle x|\Lambda_L^*(\tilde{x})\rangle\!\rangle\Big), \tag{78}$$

$$v_2 := \Big(1, 1, \langle\!\langle 0_n|\Lambda_R(\tilde{0}_n)\rangle\!\rangle, \langle\!\langle 0_n|\Lambda_R(\tilde{0}_n)\rangle\!\rangle, \langle\!\langle 0_n|\Lambda_R(\tilde{0}_n)\rangle\!\rangle, \langle\!\langle 0_n|\Lambda_R(\tilde{0}_n)\rangle\!\rangle\Big). \tag{79}$$

We can use this to upper bound the SPAM factor as

$$\begin{aligned}
\left\|\hat{\Omega}PW(\sum_{x\in\{0,1\}^n}\Theta_x)WP^\dagger\right\|_\infty &\leq \left\|\hat{\Omega}PW(\sum_{x\in\{0,1\}^n}\Theta_x)WP^\dagger\right\|_{HS} \qquad (80)\\
&= 2^n(2^n+1)^2\big[v_1 W^\dagger P^\dagger \hat{\Omega}^\dagger\hat{\Omega}PWv_1{}^\dagger\; v_2W^\dagger P^\dagger W v_2{}^\dagger\big]^{1/2}\\
&\leq 2^n(2^n+1)^2\left[\left\|\hat{\Omega}\hat{\Omega}^\dagger\right\|_\infty v_1 W^\dagger P^\dagger PWv_1{}^\dagger\; v_2W^\dagger P^\dagger W v_2{}^\dagger\right]^{1/2}\\
&\leq \frac{2^n(2^n+1)^2\left\|\hat{\Omega}\right\|_{HS}}{\big(2^n(2^{2n}-4)(2^{2n}-2)\big)^2}\Bigg[\Big[(2^{2n}-2^{2n}-2)+\mathop{\mathbb{E}}_{x\in\{0,1\}^n}\langle\!\langle x|\tilde{x}\rangle\!\rangle(4-2\,2^n)\Big]^2\\
&\qquad + 2\Big[(2-2^n)+\mathop{\mathbb{E}}_{x\in\{0,1\}^n}\langle\!\langle x|\tilde{x}\rangle\!\rangle(2^{2n}-2^n)\Big]^2\Bigg]^{1/2}\\
&\qquad \times\Bigg[\Big[(2^{2n}-2^{2n}-2)+\langle\!\langle 0_n|\tilde{0}_n\rangle\!\rangle(4-2\,2^n)\Big]^2\\
&\qquad + 2\Big[(2-2^n)+\langle\!\langle 0_n|\tilde{0}_n\rangle\!\rangle(2^{2n}-2^n)\Big]^2\Bigg]^{1/2}.
\end{aligned}$$

Gathering terms, throwing away some negative ones, remembering our bound for $\left\|\hat{\Omega}\right\|_{HS}$, and using that

$$\mathop{\mathbb{E}}_{x\in\{0,1\}^n}\langle\!\langle x|\Lambda_L^*(\tilde{x})\rangle\!\rangle \leq 1, \tag{81}$$

and $\langle\!\langle 0_n|\Lambda_R(\tilde{0}_n)\rangle\!\rangle \leq 1$ by construction we can bound this further by

$$\left\|\hat{\Omega}PW(\sum_{x\in\{0,1\}^n}\Theta_x)WP^\dagger\right\|_\infty \leq \frac{3\,2^{2n}(2^n+1)^2(2^{2n}-1)}{\big(2^n(2^{2n}-4)(2^{2n}-2)\big)^2}\Big[(2^{2n}-2^n-2)^2+2(2-2^n)^2+(2^{2n}-2^n)^2+(4-2\,2^n)^2\Big]. \tag{82}$$

We would like to stress that from this expression we can already see that the SPAM norm contribution is asymptotically independent of n. By basic numerics, we can obtain

$$\left\|\hat{\Omega}PW(\sum_{x\in\{0,1\}^n}\Theta_x)WP^\dagger\right\|_\infty \leq 11\,u(A). \tag{83}$$

We again note that this constant is sub-optimal (especially for large n). Putting all of this together we obtain the stated bound. □

When the probe super-operator is a unitary restricted to the traceless subspace ($A = P_{\mathrm{ad}}UP_{\mathrm{ad}}$ for some unitary U) then we can obtain a substantially improved bound, which hinges critically on the fact that U is a quantum channel.

Theorem 13 (Restatement of Theorem 2 in the main text)**.** *Consider the n-qubit Clifford UIRS protocol and let $A = P_{\mathrm{ad}}UP_{\mathrm{ad}}$ be a probe super-operator with U a unitary. Also let $\tilde{0}_n$ be a noisy implementation of the all-zero state and $\{\tilde{x}\}_x$ the noisy computational basis POVM). The dynamic shadow norm is bounded as*

$$\|A\|_{\mathrm{dyn},m} \leq 10. \tag{84}$$

Proof. We begin from the definition of the dynamic shadow norm, given by

$$\|A\|_{\mathrm{dyn},m} = \max_{\Lambda_R,\Lambda_L} (2^n+1)^2 2^n \sum_{x\in\{0,1\}^n} \langle\!\langle x^{\otimes 2}\otimes \tilde{x}| \Big[\mathop{\mathbb{E}}_{g\in\mathbb{C}_n} \phi(g)^{\otimes 3}(P_{\mathrm{ad}} U P_{\mathrm{ad}})^{\otimes 2}\otimes \Lambda \mathop{\mathbb{E}}_{g\in\mathbb{C}_n} \phi(g)^{\otimes 3}\Big]^m |0_n^{\otimes 2}\otimes \tilde{0}_n\rangle\!\rangle. \tag{85}$$

Here, again we write $\Lambda := \Lambda_R\Lambda_L$. Note also that $P_{\mathrm{ad}}^2 = P_{\mathrm{ad}}$ and that P_{ad} commutes with both U and $\phi(g)$. Hence, we can write

$$\|A\|_{\mathrm{dyn},m} = \max_{\Lambda_R,\Lambda_L} (2^n+1)^2 2^n \mathop{\mathbb{E}}_{x\in\{0,1\}^n} \langle\!\langle x^{\otimes 2}\otimes \Lambda_L^*(\tilde{x})|(P_{\mathrm{ad}}^{\otimes 2}\otimes \mathbb{I})\Big[\mathop{\mathbb{E}}_{g\in\mathbb{C}_n} \phi(g)^{\otimes 3}(U^{\otimes 2}\otimes \Lambda) \mathop{\mathbb{E}}_{g\in\mathbb{C}_n} \phi(g)^{\otimes 3}\Big]^m |0_n^{\otimes 2}\otimes \Lambda_R(\tilde{0}_n)\rangle\!\rangle. \tag{86}$$

Furthermore $\mathbb{E}_{g\in\mathbb{C}_n}\phi(g)^{\otimes 3}$ is a projector, and thus

$$\begin{aligned}\|A\|_{\mathrm{dyn},m} = \max_{\Lambda_R,\Lambda_L} (2^n+1)^2 2^n \mathop{\mathbb{E}}_{x\in\{0,1\}^n} \langle\!\langle x^{\otimes 2}\otimes \Lambda_L^*(\tilde{x})|(P_{\mathrm{ad}}^{\otimes 2}\otimes \mathbb{I}) \sum_{\pi,\pi'} W_{\pi,\pi'}|\pi\rangle\!\rangle\langle\!\langle \pi'| \\ \times \Big[\mathop{\mathbb{E}}_{g\in\mathbb{C}_n} \phi(g)^{\otimes 3}(U^{\otimes 2}\otimes \Lambda) \mathop{\mathbb{E}}_{g\in\mathbb{C}_n} \phi(g)^{\otimes 3}\Big]^m |0_n^{\otimes 2}\otimes \Lambda_R(\tilde{0}_n)\rangle\!\rangle,\end{aligned} \tag{87}$$

where we have used the resolution of the third-moment projector as in eq. (49). Now defining the vectors

$$\hat{v}_\pi := \langle\!\langle x^{\otimes 2}\otimes \Lambda_L^*(\tilde{x})|(P_{\mathrm{ad}}^{\otimes 2}\otimes \mathbb{I})|\pi\rangle\!\rangle, \tag{88}$$

$$\hat{w}_{\pi'}(m) := \langle\!\langle \pi'| \Big[\mathop{\mathbb{E}}_{g\in\mathbb{C}_n} \phi(g)^{\otimes 3}(U^{\otimes 2}\otimes \Lambda) \mathop{\mathbb{E}}_{g\in\mathbb{C}_n} \phi(g)^{\otimes 3}\Big]^m |0_n^{\otimes 2}\otimes \Lambda_R(\tilde{0}_n)\rangle\!\rangle, \tag{89}$$

we can express the shadow norm as

$$\|A\|_{\mathrm{dyn},m} = \max_{\Lambda_R,\Lambda_L} (2^n+1)^2 2^n v W w^T(m). \tag{90}$$

By direct calculation analogous to the calculations done in the proof of theorem 10, this becomes

$$\begin{aligned}\|A\|_{\mathrm{dyn},m} = \max_{\Lambda_R,\Lambda_L} \frac{(2^n+1)^2 2^n}{2^n(2^{2n}-4)(2^{2n}-1)} \Big[\big((2^{2n}-2) - 2 \mathop{\mathbb{E}}_{x\in\{0,1\}^n} \langle\!\langle x|\Lambda_L^*(\tilde{x})\rangle\!\rangle 2^n\big)\hat{w}_{(12)} \\ + \big(2^{2n} \mathop{\mathbb{E}}_{x\in\{0,1\}^n} \langle\!\langle x|\Lambda_L^*(\tilde{x})\rangle\!\rangle - 2^n\big)\hat{w}_{(123)} + \big(2^{2n} \mathop{\mathbb{E}}_{x\in\{0,1\}^n} \langle\!\langle x|\Lambda_L^*(\tilde{x})\rangle\!\rangle - 2^n\big)\hat{w}_{(132)}\Big].\end{aligned} \tag{91}$$

Finally, we use that $\|\pi'\|_\infty = 1$ and that $|0_n^{\otimes 2}\otimes \Lambda_R(\tilde{0}_n)\rangle\!\rangle$ is a quantum state to see that

$$\begin{aligned}|\hat{w}_{\pi'}(m)| &= |\langle\!\langle \pi'| \Big[\mathop{\mathbb{E}}_{g\in\mathbb{C}_n} \phi(g)^{\otimes 3}(U^{\otimes 2}\otimes \Lambda) \mathop{\mathbb{E}}_{g\in\mathbb{C}_n} \phi(g)^{\otimes 3}\Big]^m |0_n^{\otimes 2}\otimes \Lambda_R(\tilde{0}_n)\rangle\!\rangle| \\ &\leq \Big\| \Big[\mathop{\mathbb{E}}_{g\in\mathbb{C}_n} \phi(g)^{\otimes 3}(U^{\otimes 2}\otimes \Lambda) \mathop{\mathbb{E}}_{g\in\mathbb{C}_n} \phi(g)^{\otimes 3}\Big]^m \Big\|_{1\to 1} \\ &\leq 1,\end{aligned} \tag{92}$$

since $U^{\otimes 2}\otimes\Lambda$ and $\mathbb{E}_{g\in\mathbb{C}_n}\phi(g)^{\otimes 3}$ are quantum channels. Together with the fact that $|\mathbb{E}_{x\in\{0,1\}^n}\langle\!\langle x|\Lambda_L^*(\tilde{x})\rangle\!\rangle| \leq 1$, we get

$$\|A\|_{\mathrm{dyn},m} \leq \frac{(2^n+1)^2 2^n}{2^n(2^{2n}-4)(2^{2n}-1)} \Big[(2^{2n}-2) + 2\, 2^n\big) + \big(2^{2n}+2^n\big) + \big(2^{2n}+2^n\big)\Big] \leq 10 \tag{93}$$

for $n \geq 2$. ◻

Finally, we provide a proof of lemma 12, adapted from a very similar statement in ref. [9, Lemma 8.5].

Proof of lemma 12. We begin by bringing A into Schur normal form, i.e.,

$$A = U(D+N)U^\dagger \tag{94}$$

where D is diagonal (with the eigenvalues of A on the diagonal), N is strictly upper triangular and U is unitary. Now consider the expansion of $(D+N)^m$. Since N is strictly upper triangular, any term with more than $d-1$ factors of N must vanish. Hence, we have

$$\begin{aligned} \|A^m\| &= \left\|U(D+N)^m U^\dagger\right\| \\ &\leq \|\Lambda\|_\infty^m + \sum_{i=1}^{\min\{d-1,m\}} \binom{m}{k} \|D\|_\infty^{m-i} \|N\|_\infty^i \\ &\leq \|\Lambda\|_\infty^m + (d-1)m^{d-1} \max\left\{s(A)^{m-d+1}, s(A)^{m-1}\right\} \max\left\{ \|N\|_\infty, (\|N\|_\infty)^{d-1}\right\}, \end{aligned} \tag{95}$$

where we have used that $\binom{m}{k} \leq m^{d-1}$, Hölder's inequality, and the monotonicity of the exponential function. Now note that

$$\|N\|_\infty \leq \|D\|_\infty + \|A\|_\infty \leq 2\|A\|_\infty . \tag{96}$$

Combining this with $\|D\|_\infty = s(D) = s(A)$ we obtain the lemma statement. □

SUPPLEMENTARY NOTE 7. – PAULI-NOISE ESTIMATION

Here we analyze the Pauli-noise estimation scheme outlined in the main text. Recall that we consider sequences of the form $\mathbf{g} = (c^{-1}, p_m, \ldots, p_1, c)$, where $p_1, \ldots, p_m$ are i.i.d. randomly drawn elements from the Pauli group $\mathbb{P}_n$ and c is randomly drawn multi-qubit Clifford $\mathbb{C}_n$. For τ a traceless Hilbert-Schmidt normalized multi-qubit Pauli operator and sequence $\mathbf{g}$ we define the filter-function as

$$f_\tau(x, \boldsymbol{g}) := \alpha \langle\!\langle x|\omega(c)\omega(p_m)A_\tau \ldots A_\tau \omega(p_1)\omega(c)|0_n\rangle\!\rangle, \tag{97}$$

with $A_\tau = |\tau\rangle\!\rangle\langle\!\langle\tau|$ and $\alpha = 2^n(1+2^n)$. Without SPAM, the expected value of the single shot estimator, is given by

$$\begin{aligned} k_\tau(m) &= \alpha \mathop{\mathbb{E}}_{c\in\mathbb{C}_n} \sum_{x\in\{0,1\}^n} \langle\!\langle x^{\otimes 2}|\omega(c^{-1})^{\otimes 2}|\tau^{\otimes 2}\rangle\!\rangle \langle\!\langle\tau^{\otimes 2}|\omega(c^{\otimes 2})|0_n^{\otimes 2}\rangle\!\rangle\, \Lambda_{\tau,\tau}^{m-1} \\ &= \alpha \mathop{\mathbb{E}}_{c\in\mathbb{C}_n} \sum_{x\in\{0,1\}^n} \langle x|\,\omega(c)(\tau)\,|x\rangle^2 \,\langle 0|\,\omega(c)(\tau)\,|0\rangle^2\, \Lambda_{\tau,\tau}^{m-1} . \end{aligned} \tag{98}$$

with $\Lambda_{\tau,\tau} = \langle\!\langle\tau|\Lambda|\tau\rangle\!\rangle$. Since the Clifford group acts transitively on the traceless Pauli operators $\mathbb{P}_n^*$, we can rewrite

$$k_\tau(m) = \alpha \mathop{\mathbb{E}}_{\tau'\in 2^{n/2}\mathbb{P}_n^*} \sum_{x\in\{0,1\}^n} \langle x|\,\tau'\,|x\rangle^2 \,\langle 0|\,\tau'\,|0\rangle^2\, \Lambda_{\tau,\tau}^{m-1}. \tag{99}$$

Out of the $2^{2n}-1$ traceless Pauli-operators only 2^n-1 have non-vanishing diagonal entries (those consisting only out of local $\mathbb{I}$ and Z). The non-vanishing diagonal entries are all identical to $2^{n/2}$. Thus, using the definition of α we have

$$k_\tau(m) = \alpha \frac{2^n(2^n-1)}{2^{2n}(2^{2n}-1)} \Lambda_{\tau,\tau}^{m-1} = \Lambda_{\tau,\tau}^{m-1} . \tag{100}$$

It remains to calculate the variance associated with estimating $k_\tau(m)$, as given in (19) in the main text. We intend to prove that $k_\tau(m)$ has variance bound in $O(2^n)$. To do this first, note that

$$f_\tau(x, \boldsymbol{g})^2 = \langle\!\langle x^{\otimes 2}|\omega(c^{-1})^{\otimes 2}|\tau^{\otimes 2}\rangle\!\rangle \langle\!\langle\tau^{\otimes 2}|\omega(c)^{\otimes 2}|0_n^{\otimes 2}\rangle\!\rangle \tag{101}$$

since $P^{\otimes 2}\tau^{\otimes 2}P^{\dagger^{\otimes 2}} = \tau^{\otimes 2}$. Hence, the variance associated to the estimation can be upper bounded by

$$\begin{aligned} \mathbb{V}_\tau(m) \leq 2^{2n}(2^n+1)^2 \sum_{x\in\{0,1\}^n} \mathop{\mathbb{E}}_{c\in\mathbb{C}_n} &\langle\!\langle x^{\otimes 2}|\omega(c^{-1})^{\otimes 2}|\tau^{\otimes 2}\rangle\!\rangle \\ &\times \langle\!\langle\tau^{\otimes 2}|\omega(c)^{\otimes 2}|0_n^{\otimes 2}\rangle\!\rangle \langle\!\langle x|\omega(c^{-1})|\tau_0\rangle\!\rangle \\ &\times \langle\!\langle\tau_0|\omega(c)|0_n\rangle\!\rangle, \end{aligned} \tag{102}$$

where we have used that $\mathbb{E}_{p\in\mathbb{P}}\,\omega(p) = |\tau_0\rangle\rangle\langle\langle\tau_0|$ and the trace preservation of Λ. Noting again that $\omega(c)$ acts trivially on τ_0 and transversally on the traceless Pauli operators, we see that

$$\mathbb{V}_\tau(m) \le \frac{2^{3n}(2^n+1)^2}{2^{2n}-1}\,\langle 0_n|\tau_0|0_n\rangle^2 \sum_{\tau'\in\mathbb{P}_n^*} \langle 0_n|\tau'|0_n\rangle^4 \tag{103}$$

which becomes by the analogous argument as above

$$\mathbb{V}_\tau(m) \le \frac{2^{3n}(2^n+1)^3}{2^{3n}(2^{2n}-1)} = O(2^n), \tag{104}$$

as intended.

SUPPLEMENTARY NOTE 8. – MARGINAL CHANNEL RECONSTRUCTIONS AND CROSS-TALK TOMOGRAPHY

We here show how to employ the local Clifford UIRS protocol to get tomographic information of channel marginals. To this end, recall that with the local Clifford UIRS protocol we can efficiently estimate the quantity $3^{-|w|}\operatorname{Tr}(\Lambda P_w U P_w \Lambda)$ for any unitary channel U, where Λ is a quantum channel and P_w is the projector onto the irreducible representation of $\mathbb{C}_1^{\times n}$ labeled by the bit string w, provided $|w|$ bounded. We can introduce channel marginals Λ_k of Λ by inserting a maximally mixed state into all but the first k (out of n) inputs and tracing out all but the first k output qubits. Note that we can choose the order of the qubits arbitrarily, therefore restricting to the first k qubits does not cost any generality. For any $w \in \{0,1\}^k \times \{0\}^{n-k}$ we have

$$3^{-|w|}\operatorname{Tr}(\Lambda P_w A P_w) = 3^{-|w|}\operatorname{Tr}(\Lambda_k P_w A P_w), \tag{105}$$

which only depends on the marginal Λ_k. Now consider the k-qubit super-operator

$$S_k = \sum_{w\in\{0,1\}^k} P_w \Lambda_k P_w, \tag{106}$$

which we will refer to as the *pinched marginal* associated with the marginal Λ_k. Note that this super-operator is not necessarily a quantum channel (although it is trace preserving). One can see that the pinched marginal is composed of blocks $\Lambda_w = P_w \Lambda P_w$ which we refer to as the *unital marginals* in the main text.

We can reconstruct the pinched marginal S_k using the local Clifford UIRS protocol. To see this, consider the group of k-qubit Clifford operators $\mathbb{C}_k$. Reference [10, theorem 39] implies that

$$\frac{1}{|\mathbb{C}_k|}\sum_{C\in\mathbb{C}_k} \big((2^{2k}-1)\operatorname{Tr}(S_k\omega(C)^\dagger) - (2^{2k}-2)\big)\omega(C) = S_k \tag{107}$$

using that the Clifford group is a 2-design (on k qubits). Using the definition of S_k we see that

$$\operatorname{Tr}(S_k\omega(C)) = \sum_{w\in\{0,1\}^k} 3^{|w|}\big(3^{-|w|}\operatorname{Tr}(\Lambda_k P_w C P_w)\big). \tag{108}$$

From theorem 9 we know that we can estimate the quantities $3^{-|w|}\operatorname{Tr}(\Lambda_k P_w C P_w)$ to accuracy ϵ using $S = O(k2^{(2+2\log_2(3))k}/\epsilon^2)$ runs of the local Clifford UIRS protocol. Hence, we can reconstruct S_k to ϵ error in diamond norm using $S = O(k2^{(2+4\log_2(3))k}/\epsilon^2)$ runs. In particular, we can also construct every 'block' Λ_w of S_k with additive error in diamond norm from the same number of samples. Moreover, since the procedure we have described above is independent of which set of k qubits is considered, it follows immediately that one can reconstruct all $\binom{n}{k}$ pinched marginals associated to each set of k qubits to a global ϵ error in diamond norm using $S = O(nh(k/n)k2^{(2+4\log_2(3))k}/\epsilon^2)$ samples (where $h(k/n)$ is the binary entropy). Using $\log\binom{n}{k} \le k\log(en/k)$, we can relax the statement to guarantee ϵ-accurate recovery of all unital marginals Λ_w with $|w| = k$ in diamond norm from $O(k^2 2^{9k}/\epsilon^2)$.

SUPPLEMENTARY NOTE 9. – DETAILS ON SPAM-ROBUST CHANNEL RECONSTRUCTION

Using multi-qubit Clifford UIRS we can extract the relative average-gate fidelities that enter the tomographic reconstruction of both schemes from the output statistics of random gate-set sequences, without the need to perform different interleaved

experiments. This gives rise to an efficient and robust channel reconstruction protocol. In the multi-qubit Clifford UIRS protocol the decay rate are given as

$$p(A) = \frac{\mathrm{Tr}(A^\dagger \Lambda)}{2^{2n} - 1}. \tag{109}$$

Hence, if we assume that $A = P_{\mathrm{ad}} U P_{\mathrm{ad}}$ for a unitary channel U we see that $p(U) = (2^n F(U, \Lambda) - 1)/(2^n - 1)$, where $F(U, \Lambda)$ is the average fidelity between U and Λ. By theorem 13 and theorem 10, we can estimate using the UIRS protocol, an exponential number of average fidelities using only a polynomial number of samples (and equivalently channel queries). Furthermore, for U a Clifford unitary calculating the sequence correlation function, and, thus, the entire classical-post processing, is time and space efficient in the number of qubits.

Characterizing a quantum channels in terms of different relative average gate fidelities with unitaries can provide valuable diagnostic in itself. This can be seen as a robust gate-set or channel variant of selective state tomography [11]. Beyond this, building on the results of refs. [10, 12–14], having access to relative average fidelities is an powerful primitive for the tomographic reconstruction of channels, which we will now consider in more detail.

The first task we consider is the reconstruction of unitary (or more generally bounded Kraus rank) quantum channels—low-rank randomized benchmarking tomography. This task is vital to the characterization of calibration errors. Reference [10] establishes that given a list of estimates $[\hat{F}(C, \Lambda)]_{C \in \mathcal{A}}$ of relative average gate fidelities with respect to a randomly chosen subset $\mathcal{A}$ of Clifford unitaries, a constraint least-squares fit can reconstruct Λ provided that $|\mathcal{A}| \geq cd^2 \log(d)$. More precisely, the error of the channel estimate $\hat{\Lambda}$ in Hilbert-Schmidt norm of the Choi-states fulfills

$$\left\| J(\Lambda) - J(\hat{\Lambda}) \right\|_{HS} \leq 2^{2n} \frac{\left\| \hat{F} - F \right\|_2}{\sqrt{|S|}} \tag{110}$$

where $F := [F(C, \Lambda)]_{C \in \mathcal{A}}$ is the vector of average fidelities of length $|\mathcal{A}|$, $\hat{F}$ is an estimate of F produced through the shadow sequence protocol and the norm is the l_2 vector norm. Furthermore, the reconstruction is stable against Λ deviating from the low-rank assumption (model-mismatch) and can be formulated in different p-norms on both sides, we refer to the supplemental material of ref. [10] for details. Reference [10], however, has not analyzed the overall sampling complexity of the resulting RB tomography scheme when combined with a robust way to acquire the relative average fidelities.

The UIRS protocol can provide the missing piece. After decay fitting, we can give estimates $\hat{F}$ for the vector of fidelities F with error guarantee

$$\left\| \hat{F} - F \right\|_\infty = O\left(\sqrt{\frac{\log(2|\mathcal{A}|/\delta)}{S}} \right) \tag{111}$$

with success probability $1 - \delta$ using S samples, i.e., the size of the gate-set shadow. Using the standard relations between l_∞ and l_2 vector norms this implies we can obtain an ϵ-accurate reconstruction of Λ provided

$$S \geq C\, 2^{4n} \frac{\log(2|\mathcal{A}|/\delta)}{\epsilon^2} \tag{112}$$

with a suitable constant C. Dropping polynomial factors in n and $1/\epsilon$, we find that the total number of gate-set shadows scales as $O(2^{4n})$. Note that the number of channel invocations is bounded by the maximal sequence length times the number of sequences. This matches the scaling of the information theoretic lower bound derived in ref. [10] for the case that the average gate fidelities are measured independently. Besides the favourable scaling, the UIRS protocol has the benefit compared to, e.g., the interleaved protocol of ref. [13] that the same measurement data is used for estimating all the average fidelities.

Besides the compressive, low-rank quantum channel tomography, we can use average gate-fidelities from UIRS protocols for the tomography of a more general class of quantum channels. If Λ is a unital quantum channel then it is known [10, theorem 38] (see also ref. [12]) that it can be expressed as

$$\Lambda = \frac{1}{S} \sum_{C \in S} \left(DF(C, \Lambda) - 2^{-n} D + 1 \right) \omega(C) \tag{113}$$

with $D = 2^n(2^n + 1)(2^{2n} - 1)$, provided the set S is a unitary 2-design. We can provide a direct reconstruction for the unital channel Λ by calculating

$$\hat{\Lambda} = \frac{1}{S} \sum_{C \in S} \left(D\hat{F}(C, \Lambda) - 2^{-n} D + 1 \right) \omega(C) \tag{114}$$

where the estimates $\hat{F}(C,\Lambda)$ are again provided by the multi-qubit Clifford UIRS protocol. The accuracy of this reconstruction depends on the metric used. If we consider the Hilbert-Schmidt of the Choi state as before, we see that

$$\left\| J(\Lambda) - J(\hat{\Lambda}) \right\|_{HS} = \frac{D}{|S|} \sum_{C \in S} |F(C,\Lambda) - \hat{F}(C,\Lambda)|, \tag{115}$$

since $\|J(C)\|_{HS} = 1$ for all unitaries C. Hence, by the same argument as in the unitary case, the number of samples required scales as $O(2^{8n})$. The same argument holds for all norms for which $\|C\| = 1$, such as the diamond norm.

[1] W. Fulton and J. Harris, *Representation theory: a first course*, Vol. 129 (Springer Science & Business Media, 2013).

[2] D. Gross, K. M. R. Audenaert, and J. Eisert, Evenly distributed unitaries: on the structure of unitary designs, J. Math. Phys. **48**, 052104 (2007).

[3] H.-Y. Huang, R. Kueng, and J. Preskill, Predicting many properties of a quantum system from very few measurements, Nature Phys. **16**, 1050 (2020).

[4] L. Devroye, M. Lerasle, G. Lugosi, and R. I. Oliveira, Sub-gaussian mean estimators, (2015), arXiv:1509.05845.

[5] A. S. Nemirowski and D. B. Yudin, *Problem complexity and method efficiency in optimization* (John Wiley and Sons, 1983).

[6] G. Lugosi and S. Mendelson, Mean estimation and regression under heavy-tailed distributions: A survey, Found. Comp. Math. **19**, 1145 (2019).

[7] H. Zhu, Multiqubit Clifford groups are unitary 3-designs, Phys. Rev. A **96**, 062336 (2017).

[8] R. Bhatia, *Matrix analysis*, Vol. 169 (Springer Science & Business Media, 2013).

[9] M. M. Wolf, Quantum channels & operations: Guided tour, Lecture notes available at http://www-m5. ma. tum. de/foswiki/pub M **5** (2012).

[10] I. Roth, R. Kueng, S. Kimmel, Y.-K. Liu, Gross, J. Eisert, and M. Kliesch, Recovering quantum gates from few average gate fidelities, Phys. Rev. Lett. **121**, 170502 (2018).

[11] J. Morris and B. Dakić, Selective quantum state tomography (2019), arXiv:1909.05880.

[12] A. J. Scott, Optimizing quantum process tomography with unitary 2-designs, J. Phys. A **41**, 055308 (2008).

[13] S. Kimmel, M. P. da Silva, C. A. Ryan, B. R. Johnson, and T. Ohki, Robust extraction of tomographic information via randomized benchmarking, Phys. Rev. X **4**, 011050 (2014).

[14] S. Kimmel and Y. K. Liu, Phase retrieval using unitary 2-designs, in *2017 International Conference on Sampling Theory and Applications (SampTA)* (2017) pp. 345–349, arXiv:1510.08887.
